# Pituitary genomic expression profiles of steers are altered by grazing of high vs. low endophyte-infected tall fescue forages

**DOI:** 10.1371/journal.pone.0184612

**Published:** 2017-09-13

**Authors:** Qing Li, Raquel Hegge, Phillip J. Bridges, James C. Matthews

**Affiliations:** Department of Animal and Food Sciences, University of Kentucky, Lexington, Kentucky, United States of America; International University of Health and Welfare School of Medicine, JAPAN

## Abstract

Consumption of ergot alkaloid-containing tall fescue grass impairs several metabolic, vascular, growth, and reproductive processes in cattle, collectively producing a clinical condition known as “fescue toxicosis.” Despite the apparent association between pituitary function and these physiological parameters, including depressed serum prolactin; no reports describe the effect of fescue toxicosis on pituitary genomic expression profiles. To identify candidate regulatory mechanisms, we compared the global and selected targeted mRNA expression patterns of pituitaries collected from beef steers that had been randomly assigned to undergo summer-long grazing (89 to 105 d) of a high-toxic endophyte-infected tall fescue pasture (HE; 0.746 μg/g ergot alkaloids; 5.7 ha; *n* = 10; BW = 267 ± 14.5 kg) or a low-toxic endophyte tall fescue–mixed pasture (LE; 0.023 μg/g ergot alkaloids; 5.7 ha; *n* = 9; BW = 266 ± 10.9 kg). As previously reported, in the HE steers, serum prolactin and body weights decreased and a potential for hepatic gluconeogenesis from amino acid-derived carbons increased. In this manuscript, we report that the pituitaries of HE steers had 542 differentially expressed genes (P < 0.001, false discovery rate ≤ 4.8%), and the pattern of altered gene expression was dependent (P < 0.001) on treatment. Integrated Pathway Analysis revealed that canonical pathways central to prolactin production, secretion, or signaling were affected, in addition to those related to corticotropin-releasing hormone signaling, melanocyte development, and pigmentation signaling. Targeted RT-PCR analysis corroborated these findings, including decreased (P < 0.05) expression of *DRD2*, *PRL*, *POU1F1*, *GAL*, and *VIP* and that of *POMC* and *PCSK1*, respectively. Canonical pathway analysis identified HE-dependent alteration in signaling of additional pituitary-derived hormones, including growth hormone and GnRH. We conclude that consumption of endophyte-infected tall fescue alters the pituitary transcriptome profiles of steers in a manner consistent with their negatively affected physiological parameters.

## Introduction

*Epichloe coenophialum* is an endophytic fungus that infects most tall fescue (*Lolium arundinaceum*) pastures commonly used in animal grazing systems in the eastern half of the United States [1]. The interaction between *N*. *coenophialum* and tall fescue produces ergot alkaloids [2]. Consumption of ergot alkaloid-containing tall fescue impairs several metabolic, vascular, growth, and reproductive processes in cattle, collectively producing a clinical condition known as “fescue toxicosis” [3].

The anterior pituitary gland secretes hormones that affect control over several physiological processes altered by consumption of ergot alkaloid-containing forages, including hormones for metabolism (TSH), growth (GH), reproduction (LH, FSH), stress responses (ACTH), and lactation (prolactin) [4]. Despite these known relationships, we are unaware of reports that describe the effect of fescue toxicosis on pituitary genomic expression profiles.

The goal of the current research was to determine whether gene expression profiles differed between whole pituitaries of growing beef steers grazing pastures containing high (HE) or low (LE) amounts of toxic endophyte-infected tall fescue. We used transcriptome and targeted gene expression analyses to identify specific candidate molecules and signaling pathways responsible for the altered physiology of steers consuming HE forages. The global hypothesis tested was that consumption of endophyte-infected tall fescue would alter pituitary transcriptome profiles and that at least the pituitary genes responsible for the production and secretion of prolactin would be down-regulated and those for POMC/ACTH would be up-regulated.

## Materials and methods

### Animal model

All procedures involving animals were approved by the University of Kentucky Institutional Animal Care and Use Committee. The animal management regimen and model for steers that yielded the pituitary tissue of the present experiment have been reported. As described in detail previously [5–7], 19 beef steers (predominately Angus) were denied access to feed and water for 14 h, weighed, and subdivided into 2 groups based on BW. The steers were randomly allotted (d0) within BW group to graze either a low-toxic endophyte tall fescue-mixed pasture (LE; 5.7 ha; 0.023 μg ergot alkaloids/g; n = 9; BW = 267 ± 14.5 kg) or a high-toxic endophyte-infected tall fescue pasture (HE; 5.7 ha; 0.746 μg ergot alkaloids/g; n = 10; BW = 266 ± 10.9 kg) for an 89-d grazing period. Analysis of ergot alkaloid levels between the two pastures revealed that the HE steers were exposed to 25 and 21 times more ergovaline/ergovalinine and lysergic acid/isolysergic acid, respectively, than were the LE steers [5]. After the common 89-d grazing period on pastures, steers were slaughtered in the University of Kentucky Meat Laboratory (Lexington, KY) over a 17-day period. Throughout the slaughter period, steers continued to graze their respective treatment pastures. Details of the slaughter period and process have been reported [5].

### Sample collection and RNA preparation

Steers were stunned by captive bolt pistol and exsanguinated. Within 10 to 12 minutes of death, the whole pituitary was collected from each animal, placed in a foil pack, flash-frozen in liquid nitrogen, and stored at -80°C. Three pituitary glands (1 LE, 2 HE) were not used in the microarray analysis because of tissue damage incurred during the collection process. As a result, eight pituitaries (n = 8) for both LE and HE treatment groups were subjected to RNA analyses.

Total RNA was extracted from the whole frozen pituitary tissue using TRIzol Reagent (Invitrogen Corporation, Carlsbad, CA) following the manufacturer’s instructions. The RNA concentrations were determined using a NanoDrop ND-1000 Spectrophotometer (NanoDrop Technologies, Wilmington, DE), which revealed that all samples had an average concentration of 678 ng/μl and were of high purity with 260:280 nm absorbance ratios ranging from 1.71 to 1.91 and 260:230 nm absorbance ratios ranging from 2.08 to 2.55. The integrity of total RNA was examined by gel electrophoresis using an Agilent 2100 Bioanalyzer System (Agilent Technologies, Santa Clara, CA) at the University of Kentucky Microarray Core Facility. All RNA samples had 28S:18S rRNA absorbance ratios greater than 1.7 and RNA integrity numbers greater than 8.7.

### Microarray analysis

The custom WT Btau 4.0 Array (version 1) GeneChip (Affymetrix, Inc., Santa Clara, CA) was used [8] to investigate the effect of HE vs. LE consumption on bovine pituitary gene expression profiles. Microarray analysis was conducted according to the manufacturer’s standard protocol at the University of Kentucky Microarray Core Facility. Briefly, 3 μg of RNA for each sample was first reverse-transcribed (RT) to cDNA and then from cDNA (double-stranded) to complementary RNA (cRNA; single-stranded), which was then labeled with biotin. The biotinylated cRNAs were further fragmented and used as probes to hybridize the gene chips in the GeneChip Hybridization Oven 640 (Affymetrix), using 1 chip per RNA sample. After hybridization, the chips were washed and stained on a GeneChip Fluidics Station 450 (Affymetrix). The reaction image and signals were read with a GeneChip Scanner (GCS 3000, 7G; Affymetrix), and data were collected using the GeneChip Operating Software (**GCOS**, version 1.2; Affymetrix). The raw expression intensity values from the GCOS (i.e., 16 *.cel files from the raw methylation measurements) were imported into Partek Genomics Suite software (**PGS**, version 6.6; Partek Inc., St. Louis, MO). For GeneChip background correction, the algorithm of Robust Multichip Averaging adjusted with probe length and GC oligo contents was implemented [9, 10]. The background-corrected data were further converted into expression values using quantile normalization across all the chips and median polish summarization of multiple probes for each probe set.

All the GeneChip transcripts were annotated using the NetAffx annotation database for Gene Expression on Bovine GeneChip Array ST 1.1, provided by the manufacturer (http://www.affymetrix.com/analysis/index.affx, last accessed in March 2016, annotation file last updated in April 2014). Quality control of the microarray hybridization and data presentation was performed by MA plot on all the gene expression values and by box plot on the control probe sets on the Affymetrix chips. Pearson (Linear) Correlation generated the similarity matrix (last accessed in March 2016, Partek Genomics Suite 6.6 6.15.0422). The average correlation between any pair of the 16 GeneChips was 0.98, and all GeneChips were further analyzed. Principal component analysis (**PCA**) was performed to elucidate the quality of the microarray hybridization and visualize the general data variation among the chips (Partek, 2015). To assess treatment effects (HE vs. LE) on the relative expression of the pituitary gene transcripts, qualified microarray data were subjected to one-way ANOVA using the same PGS software. To achieve a higher degree of confidence (i.e., a more conservative approach), transcripts showing treatment effects at the significance level of P < 0.001 (false discovery rate of ≤ 4.8%) were defined as differentially expressed. These differentially expressed genes/gene transcripts (**DEGs**) were subjected to hierarchical clustering analysis using PGS software and to canonical, functional, and network pathway analyses using the Core Analysis program of Ingenuity Pathway Analysis online software (IPA, Build version 430059M, Content version 31813283; http://www.ingenuity.com [accessed in December, 2016]; Ingenuity Systems, Inc., Redwood City, CA).

All the microarray *.cel files collected by GCOS plus the GC Robust Multichip Averaging-corrected data processed by PGS software of this manuscript have been deposited in the National Center for Biotechnology Information’s Gene Expression Omnibus (**GEO**; http://www.ncbi.nlm.nih.gov/geo/) [released October 23, 2014]), are minimum information about a microarray experiment (**MIAME**) compliant [11], and are accessible through GEO series accession number GSE62570.

### Real-time RT-PCR analysis

Primer sets for genes selected for real-time reverse transcription (RT) PCR analysis (S1 Table) were designed using the NCBI Pick Primers online program against RefSeq sequences (accessed January to June 2016). Real-time RT-PCR was performed using an Eppendorf Mastercycler ep *realplex*2 system (Eppendorf, Hamburg, Germany) with iQ SYBR Green Supermix (Bio-RAD, Hercules, CA), as described [12]. Briefly, cDNA was synthesized using the SuperScript III 1st Strand Synthesis System (Invitrogen), with 1 μg of RNA used for each reverse transcription reaction. Real-time RT-PCR was performed with a total volume of 25 μL per reaction, with each reaction containing 5 μL of cDNA, 1 μL of a 10 μM stock of each primer (forward and reverse), 12.5 μL of 2× SYBR Green PCR Master Mix, and 5.5 μL of nuclease-free water. Gene expression was analyzed by the 2^−ΔΔCT^ method [13].

The resulting real-time RT-PCR products were purified using a PureLink Quick Gel Extraction Kit (Invitrogen) and sequenced at Eurofins Scientific (Eurofins, Louisville, KY). Sequences were compared with the corresponding RefSeq mRNA sequences used as the templates for primer set design. The sequences of the primers and the resulting sequence-validated real-time RT-PCR reaction amplicons for selected DEGs and the endogenous control genes *ACTB*, *PPIA*, and *UBC* are presented in S1 Table and S1 Fig, respectively. Primers for *ACTB* were from Lisowski et al. [14], and primers for *s-PRLR* and *l-PRLR* were from Thompson et al. [15]. All sequenced amplicons had at least 98% identity with their template sequences. The raw CT values of *ACTB*, *PPIA*, and *UBC* in pituitary tissue of HE and LE steers did not differ (P = 0.57, 0.42, 0.82; respectively). Accordingly, the geometric mean expression of *ACTB*, *PPIA*, and *UBC* was used to normalize the relative quantities of the selected DEGs mRNA expression, and all RT-PCR reactions were conducted in triplicate.

### Selected miRNA-target gene interactions

To identify (predict) microRNAs (miRNAs) that might regulate [15, 16] prolactin or POMC/ACTH production, microarray-identified differentially expressed miRNAs (**DEMs**) were uploaded into TargetScan online software (Release 7.1, http://www.targetscan.org/), and the species-specific “Cow” filter applied. The resulting miRNA candidates were ranked based on cumulative weighted context++ scores [16] and then reduced to only those predicted to bind mRNA of genes involved in prolactin or POMC/ACTH production or to bind to mRNA coding known transcription factors of prolactin and POMC/ACTH pathway genes.

### Statistical analyses

To test for HE vs. LE treatment effects on the relative expression of the pituitary gene transcripts, microarray hybridization data were subjected to one-way ANOVA using the PGS software as described in the “Microarray Analysis” section above. To determine the effect of treatment, the relative expression levels of selected DEGs analyzed by real-time RT-PCR were subjected to one-way ANOVA using the GLM procedure of the SAS statistical software package (version 9.4; SAS Inst., Inc., Cary, NC), with the endophyte level as the fixed effect. For these data, significance was declared when P ≤ 0.05, and a tendency to differ was declared when 0.10 ≥ P > 0.05.

## Results

### Differentially expressed genes

Principal component analysis of all microarray data was performed to examine the correlation and variation among the chips, revealing a total variance of 30.9% (S2 Fig). The first principal component (PC #1, x-axis) included genes with a median degree of variance (12.3%), whereas PC #2 (y-axis) and PC #3 (z-axis) encompassed genes that had low ranges of variance (9.84% and 8.75%, respectively). Overall, PCA clearly demonstrated that the chips within each treatment group were clustered closely together.

Individual ANOVA was conducted to identify altered expression of RNA transcripts in the pituitary tissue of HE vs. LE steers. At the P < 0.01 level and a false discovery rate of < 16%, 1,715 gene transcripts were identified. To refine this analysis, genes with the criteria of a false discovery rate of less than 4.8% and P < 0.001 were considered to be DEGs (S2 Table). Of these 542 DEGs, 227 (10 non-annotated) were up-regulated, 5.5% to 79.8%, and 315 (14 non-annotated) were down-regulated, 5.7% to 69.0%, in HE vs. LE steers.

Hierarchical cluster analysis of the 542 DEGs revealed that all steers were clearly separated into either the LE or HE treatment group (S3 Fig). Relative to LE steers, approximately 40% of the genes in the HE steers were up-regulated and 60% down-regulated.

### Functional, canonical pathway, and gene network analyses

To determine the physiological significance of HE-induced DEGs (S2 Table), bioinformatic analysis of canonical, functional, and network pathway analyses was performed. Canonical pathway analysis revealed (P < 0.001) that the top 7 pathways were the following: axonal guidance signaling (26 genes), role of NFAT in cardiac hypertrophy (16 genes), P2Y purigenic receptor signaling pathway (13 genes), cardiac hypertrophy signaling (17 genes), Tec kinase signaling (14 genes), ErbB signaling (10 genes), and CXCR4 signaling (13 genes) (Table 1). Additionally, several affected pathways central to prolactin production, secretion, or signaling were identified (Table 2), including dopamine receptor signaling, Gαi signaling, cAMP–mediated signaling, protein kinase A signaling, and prolactin signaling. Moreover, canonical pathway analysis also identified affected pathways involved in the signaling of other pituitary-derived hormones (Table 3): corticotropin-releasing hormone signaling, melanocyte development and pigmentation signaling, growth hormone signaling, and GnRH signaling.

**Table 1:** Top seven IPA-identified canonical pathways of genes differentially expressed by pituitary tissue of steers grazing high (HE) vs. low (LE) endophyte-infected forages.

Canonical Pathway	Number^1^	Gene Symbol	Ratio^2^	-log (P-value)
Axonal Guidance Signaling	26	*ITSN1*,*BDNF*,*PIK3R1*,*UNC5B*,*GNB5*,*ABLIM1*,*SEMA4C*,*PLCD1*,*GNB4*,*SRGAP2*,*ABLIM2*,*ACE*,*GNG12*,*PRKCA*,*EPHA7*,*PLXNC1*,*PRKCQ*,*FES*,*PAK6*,*FGFR1*,*ITGA2*,*GNG3*,*PLCL2*,*WIPF1*,*SEMA3C*,*PRKAR1A*	0.06	5.38
Role of NFAT in Cardiac Hypertrophy	16	*MAP2K6*,*PRKCQ*,*FGFR1*,*PIK3R1*,*SLC8A3*,*GNB5*,*GNG3*,*PLCL2*,*PLCD1*,*GNB4*,*MAPK10*,*RCAN3*,*ADCY8*,*GNG12*,*PRKAR1A*,*PRKCA*	0.08	5.33
P2Y Purigenic Receptor Signaling Pathway	13	*PRKCQ*,*FGFR1*,*PIK3R1*,*CREB3*,*GNB5*,*GNG3*,*PLCL2*,*PLCD1*,*GNB4*,*ADCY8*,*GNG12*,*PRKCA*,*PRKAR1A*	0.09	5.04
Cardiac Hypertrophy Signaling	17	*MAP2K6*,*DIRAS3*,*PIK3R1*,*FGFR1*,*IL6R*,*GNB5*,*GNG3*,*MAP3K5*,*PLCL2*,*PLCD1*,*GNB4*,*RHOQ*,*MAPK10*,*ADCY8*,*MAP3K3*,*GNG12*,*PRKAR1A*	0.07	4.80
Tec Kinase Signaling	14	*GNB4*,*PRKCQ*,*RHOQ*,*PAK6*,*DIRAS3*,*PIK3R1*,*FGFR1*,*ITGA2*,*GNB5*,*MAPK10*,*GNG3*,*FRK*,*GNG12*,*PRKCA*	0.08	4.77
ErbB Signaling	10	*GNB4*,*PRKCQ*,*RHOQ*,*PAK6*,*DIRAS3*,*PIK3R1*,*FGFR1*,*GNB5*,*MAPK10*,*GNG3*,*ADCY8*,*GNG12*,*PRKCA*	0.10	4.41
CXCR4 Signaling	13	*GNB4*,*PRKCQ*,*RHOQ*,*PAK6*,*DIRAS3*,*PIK3R1*,*FGFR1*,*GNB5*,*MAPK10*,*GNG3*,*ADCY8*,*GNG12*,*PRKCA*	0.08	4.14

^1^The number of genes (listed in the “Symbol” column) associated with the particular canonical pathway.

^2^The ratio is calculated as the number of genes in a given pathway that meet cutoff criteria (e.g., the ANOVA P-value for the differential expression between HE and LE groups is less than 0.001) divided by the total number of genes that make up that pathway.

**Table 2:** IPA-identified canonical pathways of genes central to prolactin production, secretion, or signaling differentially-expressed by pituitary tissue of steers grazing high (HE) vs. low (LE) endophyte-infected forages.

Canonical Pathway	Number^1^	Gene Symbol	Ratio^2^	-log (P-value)
Dopamine Receptor Signaling	4	*PRL*,*ADCY8*,*DRD2*,*PRKAR1A*	0.04	0.91
Gαi Signaling	9	*GABBR2*,*GNB4*,*GNB5*,*HTR1F*,*GNG3*,*ADCY8*,*DRD2*,*GNG12*,*PRKAR1A*	0.07	2.95
cAMP-mediated Signaling	9	*PDE8A*,*GABBR2*,*PKIB*,*CREB3*,*HTR1F*,*ADCY8*,*DRD2*,*CNGA3*,*PRKAR1A*	0.04	1.31
Protein Kinase A Signaling	20	*PRKCQ*,*PTPRD*,*CREB3*,*MYLK3*,*GNB5*,*GNG3*,*PLCL2*,*CNGA3*,*PDE8A*,*PLCD1*,*GNB4*,*DUSP10*,*ADCY8*,*PTPRN*,*EYA1*,*KDELR2*,*GNG12*,*TCF7L2*,*PRKCA*,*PRKAR1A*	0.05	3.45
Prolactin Signaling	6	*PRKCQ*,*PRL*,*PIK3R1*,*FGFR1*,*PRLR*,*PRKCA*	0.07	2.04

^1^The number of genes (listed in the “Symbol” column) associated with the particular canonical pathway.

^2^The ratio is calculated as the number of genes in a given pathway that meet cutoff criteria (e.g., the ANOVA P-value for the differential expression between HE and LE groups is < 0.001) divided by the total number of genes that make up that pathway.

**Table 3:** IPA-identified canonical pathways of genes involved in signaling of selected pituitary-derived hormones differentially-expressed by pituitary tissue of steers grazing high (HE) vs. low (LE) endophyte-infected forages.

Canonical Pathway	Number^1^	Gene Symbol	Ratio^2^	-log (P-value)
Melanocyte Development and Pigmentation Signaling	7	*PIK3R1*,*FGFR1*,*CREB3*,*POMC*,*RPS6KA5*,*ADCY8*,*PRKAR1A*	0.07	2.38
Corticotropin-releasing Hormone Signaling	7	*PRKCQ*,*BDNF*,*CREB3*,*POMC*,*ADCY8*,*PRKCA*,*PRKAR1A*	0.06	1.87
Growth Hormone Signaling	6	*IGF2*,*PRKCQ*,*PIK3R1*,*FGFR1*,*RPS6KA5*,*PRKCA*	0.07	2.06
GnRH Signaling	10	*MAP2K6*,*PRKCQ*,*PAK6*,*CREB3*,*MAPK10*,*MAP3K5*,*ADCY8*,*MAP3K3*,*PRKCA*,*PRKAR1A*	0.07	3.26

^1^The number of genes (listed in the “Symbol” column) associated with the particular canonical pathway.

^2^The ratio is calculated as the number of genes in a given pathway that meet cutoff criteria (e.g., the ANOVA P-value for the differential expression between HE and LE groups is less than 0.001) divided by the total number of genes that make up that pathway.

To refine this analysis to pituitary-specific metabolism, IPA analysis was re-run after applying the pituitary gland-specific filter. Diseases and Bio Function Analysis found (P ≤ 0.01) putative changes in diseases and disorders, molecular and cellular functions, and physiological system development and function, resulting from the differential expression of 5 genes (*DRD2*, *PRL*, *ESR1*, *POMC*, and *TCF7L2*).

**Fig 1:**
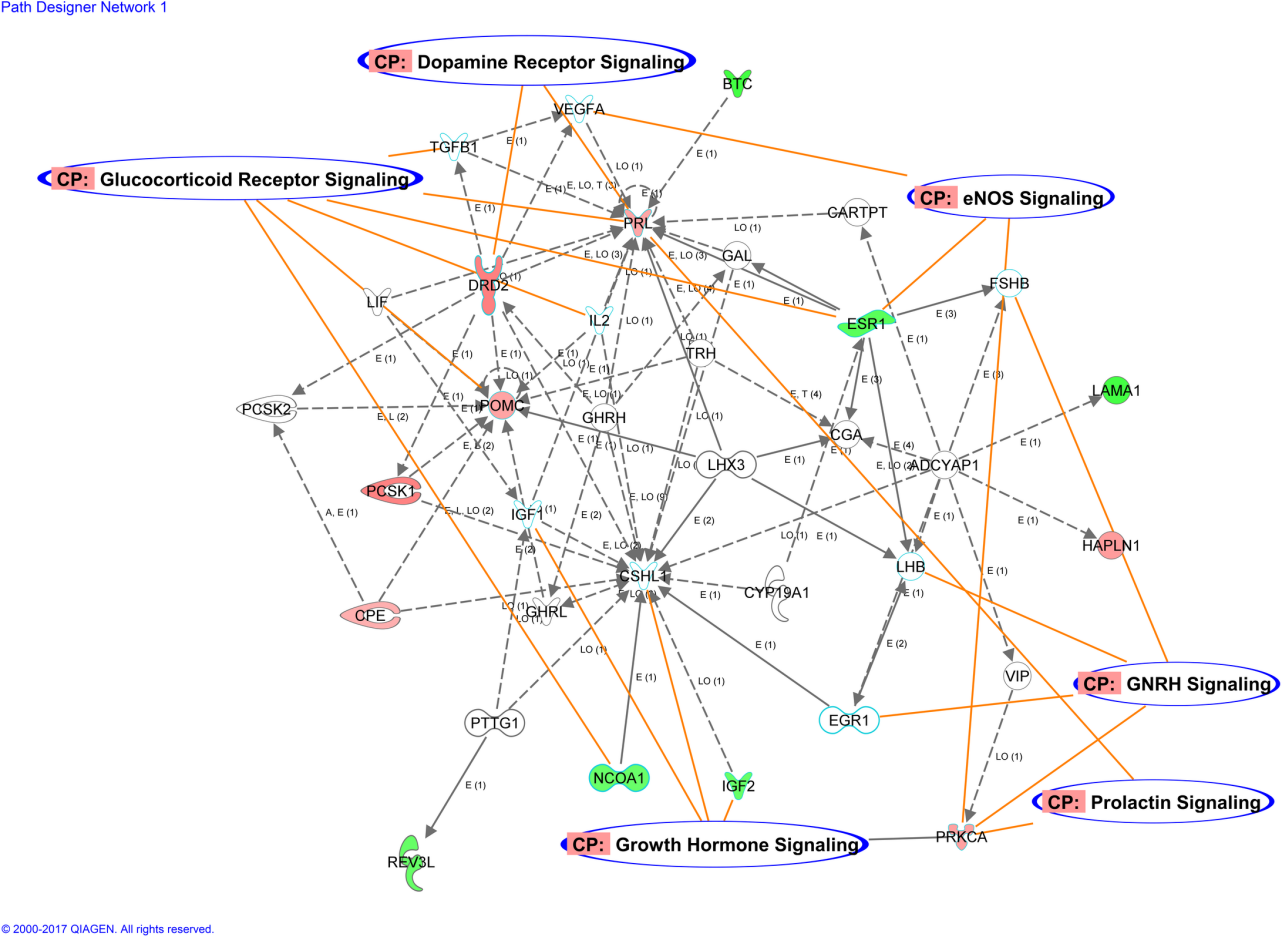
Canonical pathway network analysis. The red or green coloring represents down- or up-regulation, respectively, whereas no color indicates the molecule was added from the Ingenuity Knowledge Base (Ingenuity Pathway, Ingenuity Systems, Inc., Redwood City, CA). The intensity of the node color (light to dark) proportionally indicates the degree of differential expression. Straight lines represent binding only, whereas arrowheads symbolize action-on. A crosshead bar signifies inhibition. Labels of interaction or relationship: A = Activation, CP = Canonical Pathway, E = Expression (includes metabolism or synthesis for chemicals), I = Inhibition, LO = Localization. The number in parenthesis for each interaction indicates the number of published references in the Ingenuity Knowledge Base that support the particular interaction.

To gain insight into potentially interacting canonical pathways, pathway network analysis revealed one network that included 13 DEGs (*BTC*, *CPE*, *DRD2*, *ESR1*, *HAPLN1*, *IGF2*, *LAMA1*, *NCOA1*, *PCSK1*, *POMC*, *PRKCA*, *PRL*, and *REV3L*). Overlaying of canonical pathways revealed cross talk among several cell signaling pathways (Fig 1), including glucocorticoid receptor signaling (*ESR1*, *IL2*, *NCOA1*, *POMC*, *PRL*, *TGFB1*), GnRH signaling (*EGR1*, *FSHB*, *LHB*, *PRKCA*), growth hormone signaling (*CSHL1*, *IGF1*, *IGF2*, *PRKCA*), eNOS signaling (*ESR1*, *PRKCA*, *VEGFA*), dopamine receptor signaling (*DRD2*, *PRL*), and prolactin signaling (*PRKCA*, *PRL*).

### Real-time reversed-transcribed PCR analysis of selected mRNA

Real-time RT-PCR analysis was used to corroborate the microarray analysis-identified altered expression of key genes responsible for prolactin synthesis and secretion and POMC/ACTH production in HE vs. LE steers (Table 4). The results of these two analyses were consistent for all the targeted genes, with the exception of *PRLR*, although the statistical significance (ANOVA P-value) and fold changes measured by the two analytical techniques differed for some genes. For *PRLR*, unlike the microarray analysis, RT-PCR analysis was designed to delineate the long form (*l-PRLR*) and short form (*s-PRLR*). In the microarray analysis, *PRLR* was down-regulated in HE steers (P < 0.001), whereas in RT-PCR analysis, expression of *s-PRLR* was not altered (P = 0.21) and expression of *l-PRLR* had a tendency to differ (P < 0.07) in HE vs. LE steers.

**Table 4:** Comparison of microarray and real-time RT-PCR identification of selected genes by pituitary tissue of steers grazing high (HE) vs. low (LE) endophyte-infected forages.

Gene	Gene Name	Microarray			Real-time RT-PCR		
		Change^2^	Ratio^3^	P-value	Change^2^	Ratio^3^	P-value
*ACTB*^*1*^	Actin, beta	1.03	1.03	0.084	1.01	1.01	0.568
*PPIA*^*1*^	Peptidylprolyl isomerase A	-1.07	0.93	0.441	1.00	1.00	0.422
*UBC*^*1*^	Ubiquitin C	1.00	1.00	0.994	1.00	1.00	0.816
*DRD2*	Dopamine receptor D2	-1.76	0.57	0.001	-2.14	0.47	0.001
*PRL*	Prolactin	-1.23	0.81	0.001	-5.67	0.18	0.001
*PRLR*	Prolactin receptor	-1.31	0.76	0.001	NA	NA	NA
*s-PRLR*	Prolactin receptor short isoform	NA	NA	NA	-1.20	0.83	0.210
*l- PRLR*	Prolactin receptor long isoform	NA	NA	NA	-1.29	0.78	0.062
*POU1F1*	POU class 1 homeobox 1	-1.30	0.77	0.003	-1.47	0.68	0.038
*GAL*	Galanin/GMAP prepropeptide	-1.34	0.74	0.009	-2.35	0.43	0.019
*VIP*	Vasoactive intestinal peptide	-1.76	0.57	0.003	-2.08	0.48	0.045
*POMC*	Proopiomelanocortin	-1.25	0.80	0.001	-2.27	0.44	0.006
*PCSK1*	Proprotein convertase subtilisin/kexin type 1	-1.72	0.58	0.001	-2.02	0.5	0.001
*GH1*	Growth Hormone 1	1.01	1.01	0.728	1.20	1.20	0.436
*TSHB*	Thyroid stimulating hormone beta	1.00	1.00	0.999	1.14	1.14	0.418
*TBX19*	T-Box 19	-1.14	0.88	0.104	-1.20	0.84	0.217
*NeuroD1*	Neuronal differentiation 1	1.19	1.19	0.178	1.18	1.18	0.415
*NR3C1*	Nuclear receptor subfamily 3 group C member 1	1.14	1.14	0.112	1.33	1.33	0.270
*CRHR1*	Corticotropin-releasing hormone receptor 1	1.19	1.19	0.106	1.39	1.39	0.192

^1^Expression reference genes.

^2^Data are expressed as fold change in HE relative to LE expression.

^3^Data are expressed as ratio of HE relative to LE expression.

Although microarray analysis did not identify them as DEGs (S3 Table), the expression of 3 genes was assessed by RT-PCR analyses because they are known targets of POU1F1 (*GH1*, *TSHB*) or are involved in CRH stimulation of ACTH production (*CRHR1*). RT-PCR analysis corroborated the microarray analysis that pituitary expression of these genes did not differ between HE and LE steers (Table 4).

### Differentially expressed miRNAs (DEMs) and their predicted target genes associated with prolactin and POMC/ACTH production

The microarray chips used for this study detected 574 miRNAs. Of these, only 6 were differentially expressed (P < 0.001) in HE vs. LE steers (S2 Table). Specifically, miR-380 (42%), miR-2318 (17%), miR-329B (36%), and miR-544A (38%) were down-regulated in HE vs. LE steers, whereas miR-2356 (38%) and miR-2400 (8%) were up-regulated. The target genes of these DEMs that were associated with prolactin or POMC/ACTH production are listed in Table 5. Although no miRNAs known to directly target mRNA for prolactin were differentially expressed, every DEM targeted multiple prolactin transcription factors, stimulators, and (or) inhibitors, including miR-544A that targeted all the *PRL*-associated genes. Overall, the mRNA for three transcription factors (*POULF1*, *ESR1*, *PREB*), two transcription stimulators (*EGF*, *IKZF1*), and one transcription inhibitor (*PKIA*) of *PRL* were predicted to be targets of the DEMs. With specific regard to microarray-identified DEGs (Table 4) targeted by DEMs, *PRLR* was predicted to be the target of five DEMs and *ESR1* the target of four DEMs.

**Table 5:** Predicted relationship between differentially-expressed mRNA of prolactin and ACTH pathway genes, including transcription factors (TF), transcription stimulators (TS), and transcription inhibitors (TI), known to be targets of microarray-identified differentially-expressed miRNAs (DEMs)^1^.

Gene Symbol	Gene Description	DEM (P < 0.001)^2^^,^^3^
*PRL*	Prolactin	
*PRLR*	Prolactin Receptor	miR-329B, miR-380, miR-544A, miR-2318, miR-2356
*DRD2*	Dopamine Receptor D2	
*POU1F1* (TF for *PRL*)	POU Class 1 Homeobox 1	miR-544A
*VIP*	Vasoactive Intestinal Peptide	miR-544A, miR-2400
*ESR1* (TF for *PRL*)	Estrogen Receptor 1	miR-329B, miR-380, miR-544A, miR-2356
*PREB* (TF for *PRL*)	Prolactin Regulatory Element Binding	miR-544A, miR-2400
*EGF* (TS for *PRL*)	Epidermal Growth Factor	miR-380, miR-544A, miR-2356
*IKZF1* (TS for *PRL*)	IKAROS Family Zinc Finger 1	miR-380, miR-544A, miR-2400
*PKIA* (TI for *PRL*)	CAMP-Dependent Protein Kinase Inhibitor Alpha	miR-329B, miR-380, miR-544A, miR-2318, miR-2356, miR-2400
*POMC*	Proopiomelanocortin	
*PCSK1*	Proprotein Convertase Subtilisin/Kexin Type 1	miR-380
*TBX19* (TF for *POMC*)	T-Box 19	miR-380
*NEUROD1* (TF for *POMC*)	Neuronal Differentiation 1	miR-380, miR-544A, miR-2318
*JUN* (TF for *POMC*)	Jun Proto-Oncogene, AP-1 Transcription Factor Subunit	miR-2400
*LEP* (TS for *POMC*)	Leptin	miR-544A
*LIF* (TS for *POMC*)	Leukemia Inhibitory Factor	miR-2400
*NR3C1* (TI for *POMC*)	Nuclear Receptor Subfamily 3 Group C Member 1	miR-380, miR-544A, miR-2318, miR-2356
*SMARCA4* (TI for *POMC*)	SWI/SNF Related, Matrix Associated, Actin Dependent Regulator Of Chromatin, Subfamily A, Member 4	miR-329B
*HDAC2* (TI for *POMC*)	Histone Deacetylase 2	miR-380, miR-2356

^1^Putative gene targets of DEMs were identified using TargetScan (Release 7.1, http://www.targetscan.org).

^2^miR-329B: GenBank accession number is NR_031209 and is known as miR-329 by TargetScan.

^3^miR-544A: GenBank accession number is NR_031187 and is known as miR-544 by TargetScan.

Analogously, for POMC/ACTH production genes, whereas no miRNAs were differentially expressed that targeted *POMC* per se, TargetScan predicted that DEMs would interact with the mRNA of three transcription factors (*TBX19*, *NEUROD1*, *JUN*), two transcription stimulators (*LEP*, *LIF*), and three transcription inhibitors (*NR3C1*, *SMARCA4*, *HDAC2*) of the POMC production pathway. With specific regard to microarray-identified DEGs targeted by DEMs, *PCSK1* was the target of a single miRNA (miR-380).

Because *POMC* expression was altered and miR-380 is predicted to target two *POMC* transcription factors (*NEUROD1*, *TBX19*), the expression of *NEUROD 1* and *TBX19* was evaluated by RT-PCR, although their expression was not altered as determined by microarray analysis (S3 Table). However, consistent with the microarray analysis, the expression of *NEUROD 1* and *TBX19* based on RT-PCR analysis was not altered (Table 4).

Although the expression of *NR3C1* was not affected based on microarray analysis (S3 Table), the expression was evaluated by RT-PCR because glucocorticoid receptor complex represses the *POMC* gene through a negative glucocorticoid response element of *POMC* promoter [17]. However, RT-PCR analysis found no difference in *NR3C1* abundance in the pituitaries of HE and LE steers (Table 4).

## Discussion

The pituitary is an endocrine gland composed of anterior, intermediate, and posterior lobes, with the anterior lobe occupying approximately 80% of the entire gland. The anterior lobe is composed of five tropic cell types, which together secrete six hormones: corticotrophs (ACTH), gonadotrophs (FSH and LH), lactotrophs (prolactin), somatotrophs (GH), and thyrotrophs (TSH). Previous studies show that hormone production by all five anterior pituitary cell types is affected by the consumption of ergot alkaloids in cattle [18, 19], with decreased concentrations of serum prolactin one of the most common serological signs [20, 21].

To our knowledge, the effect of ergot alkaloid consumption on pituitary transcriptomic profiles has not been reported. To obtain this information, we conducted transcriptome analysis of pituitaries collected from previously described [5] beef steers suffering from fescue toxicosis induced by summer-long grazing (89 to 105 d) of HE and LE pastures. Importantly, concentrations of prolactin in the serum of HE steers were only approximately 10% of those of the LE steers [5], and the glucocorticoid receptor-mediated pathway was implicated in observed changes in carbohydrate metabolism in HE steers [6]. As noted in the Introduction, the goal of the current research was to determine whether gene expression profiles differed between whole pituitaries of HE and LE steers using transcriptome and targeted gene expression analysis and to identify specific candidate molecules and signaling pathways responsible for the altered physiology of steers consuming ergot alkaloid-containing tall fescue. The global hypothesis tested was that consumption of endophyte-infected tall fescue would alter pituitary transcriptome profiles. At the P < 0.001 level, the microarray analysis approach revealed the differential expression of 542 RNA transcripts by the pituitary. Importantly, the pattern of altered gene expression was clearly dependent on treatment according to hierarchical cluster analysis (S3 Fig). Thus, the first salient finding of this study is that summer-long grazing of endophyte-infected tall fescue alters the pituitary transcriptome; thus, the global hypothesis is accepted.

More specifically, given that the serum prolactin concentrations of HE steers were only approximately 10% of those of the LE steers [5], and that the glucocorticoid receptor-mediated pathway was implicated in observed changes in carbohydrate metabolism in HE steers [6], the expectation was that the expression pattern for pituitary genes responsible for the production and secretion of prolactin would be consistent with a down-regulated capacity, whereas that for POMC/ACTH would be consistent with an up-regulated capacity. Conclusions reached about these hypotheses, as well as the possible roles of miRNA in these processes, are discussed below.

### Fescue toxicosis and prolactin synthesis and secretion

The effect of ergot alkaloid consumption on prolactin production and secretion is best understood through the interactive pathway of dopamine receptors located on the surface of lactotrophs. Dopamine is one of the most influential regulators of prolactin secretion. Activation of the dopamine receptor suppresses *PRL* gene expression via the inhibition of adenylyl cyclase and prolactin exocytosis through modification of several potassium and calcium channels [22]. One way by which ergot alkaloid consumption directly affects lactotrophs is through the binding and stimulation of dopamine type two receptors (*DRD2*) on the cell surface [22]. Ergot alkaloids ingested with consumption of endophyte-infested tall fescue structurally resemble various biogenic amines, such as dopamine [3]. These ergot amines can bind to dopamine type two receptors, stimulate the receptors, and reduce basal level prolactin production and secretion as described above [23]. Consistent with this understanding, the HE steers in this study had serum prolactin concentrations that were only 10% of those of the LE steers [5]. The lower prolactin found in serum of steers exposed to HE pasture directly corresponded to the microarray and real-time RT-PCR results regarding the gene expression of *DRD2*, *POU1F1* (a.k.a. Pit1), *PRL* and *PRLR* genes (Table 4). Based on real-time RT-PCR results, the expression of these genes decreased by approximately 53%, 32%, 82%, and 22% (long isoform of prolactin receptor with tendency to differ), respectively, in HE vs. LE steers. *POU1F1* plays a pivotal role in *PRL* expression by binding to specific sites of promoter elements in the *PRL* gene [24]. Therefore, decreased expression of *POU1F1* might explain reduced *PRL* mRNA expression in HE steers to a certain extent.

An apparently associated finding was the accompanying down regulation of both *DRD2* and *PRLR* genes. Although speculative, a decrease in the expression of *DRD2* may have been a preventive measure by the lactotrophic cells to counteract the suppression of prolactin production due to the activation of the dopamine receptors, whereas the down regulation of prolactin receptor mRNA in pituitary tissue may be the result of a decreased requirement for prolactin binding in a prolactin-poor environment. Additionally, expression of *GAL* (galanin/GMAP prepropeptide) and *VIP* (vasoactive intestinal peptide) also decreased in HE vs. LE steers according to both microarray and real-time RT-PCR results (Table 4). Galanin is known to stimulate prolactin release [25, 26], although the mechanism has not been clearly defined. Additionally, galanin may directly stimulate prolactin expression and act as a lactotroph growth factor, particularly when exposure to estrogen is high [26]. Vasoactive intestinal peptide also stimulates prolactin secretion in multiple species, with receptors found on lactotrophs [27–30]. Although the mechanism by which vasoactive intestinal peptide stimulates prolactin release is not well delineated, as for galanin, cAMP accumulation and a delayed increase in calcium concentration were observed in the process [30, 31]. Thus, our hypothesis that at least the pituitary genes responsible for the production and secretion of prolactin would be down-regulated is also accepted.

In addition to prolactin, *POU1F1* activates growth hormone (*GH1*) promoter transcriptionally [32] and is involved in thyrotropin-releasing hormone (TRH) stimulation of the beta subunit of thyroid-stimulating hormone (*TSHB*) expression [33]. However, RT-PCR (Table 4) analysis corroborated the microarray (S3 Table) findings that neither *GH1* nor *TSHB* was differentially expressed in HE vs. LE steers.

Although best known for the role in regulating lactation, prolactin affects a wide variety of biological functions [34, 35], including reproduction, osmoregulation, antiangiogenic activity, regulation of immune responses, regulation of insulin release, and control of growth. With regard to growth, prolactin is associated with food intake and body weight and may interact with hypothalamic neurons responsible for appetite regulation [36, 37]. Moreover, as described in detail previously [5], the average daily gain of HE steers was 31% less than that of LE steers (P < 0.05), and the final body weight of HE steers was 7.4% less than that of LE steers (P < 0.05). Hence, reduced prolactin concentrations in HE steers might account for these observations to a certain degree.

### Fescue toxicosis, POMC/ACTH synthesis, and gluconeogenesis

As noted in the Introduction, increased mitochondrial mass and capacity for ATP synthesis and amino acid-derived gluconeogenesis [5] are postulated to be coordinated through the glucocorticoid receptor-mediated pathway [6]. Therefore, a reasonable hypothesis is that the capacity for glucocorticoid synthesis (POMC/ACTH production) would be elevated in the pituitaries of HE vs. LE steers. However, although we did not measure ACTH stimulation of cortisol release by the adrenal glands, canonical pathway analysis of pituitary DEGs indicated (z-score less than -2.00) the down-regulation of the corticotropin-releasing hormone (CRH) signaling pathway (*BDNF*, *POMC*, *ADCY8*, *PRKCA*, and *PRKAR1A*) in HE steers (Table 3). As part of the hypothalamic–pituitary–adrenal axis, the primary function of CRH is to stimulate ACTH production from the pituitary through interaction with CRHR1, the predominant pituitary-expressed CRH receptor. According to the microarray and RT-PCR analyses (S3 Table, Table 4), *CRHR1* mRNA expression level was not affected in HE vs. LE steers, whereas *CRHR2* was not qualified for RT-PCR analysis because of the low expression level. These findings are consistent with the understanding that *CRHR1* is highly expressed by the pituitary, whereas *CRHR2* is predominately expressed by brain and peripheral tissues [38]. ACTH is synthesized within the anterior pituitary as part of the much larger precursor molecule proopiomelanocortin (POMC), which is cleaved into smaller peptide hormones in a tissue-specific manner by proprotein convertases. In pituitary corticotrophs, proprotein convertase 1 (encoded by the *PCSK1* gene) alone is expressed and cleaves POMC, producing ACTH, β-endorphin, β-lipotrophin, amino-terminal peptide, and joining peptide [39]. According to the microarray and real-time RT-PCR analyses (Table 4), the abundance of both *POMC* and *PCSK1* mRNA was reduced in the pituitaries of HE vs. LE steers. Thus, the hypothesis that expression of pituitary genes responsible for the production of POMC/ACTH would be increased is rejected.

Despite the importance to adrenal steroidogenesis, research describing the effects of fescue toxicosis on blood ACTH is lacking. Moreover, although studies have been conducted to better understand the relationship between fescue toxicosis and circulating cortisol in cattle, their results are discordant [40–42]. To resolve the apparent enigma that HE steers displayed a reduced potential for pituitary synthesis of ACTH (this study), yet increased hepatic gluconeogenesis capacity [5, 6], further research is required.

### Role of miRNAs in regulating prolactin and POMC/ACTH pathways

Messenger RNA abundance is regulated by a combination of pre-transcription and post-transcription events. Transcription factors contribute to mRNA abundance at the pre-transcription level by binding to DNA and either positively or negatively regulating gene transcription [43]. MicroRNAs regulate mRNA abundance at the post-transcriptional level through complementary binding of target mRNA transcripts, resulting in repressed translation or enhanced degradation of bound mRNA [44]. Thus, decreased expression of a given miRNA would result in increased target mRNA abundance and vice versa. miR-544A, which putatively regulates multiple transcription factors and stimulators (*ESR1* [45], *EGF* [46], *IKZF1* [47], *POU1F1* [48], *PREB* [49], *VIP* [50]) of the prolactin gene (Table 5), was down-regulated 38% in HE vs. LE steers; however, expression of *PRL* decreased in HE vs. LE steers. Inconsistency between the abundance of miR-380 and its target gene was also found. Because miR-380 is predicted to target *PCSK1* and two *POMC* transcription factors, *NEUROD1* [51] and *TBX19* [52] (Table 5), we expected the decrease in expression of miR-380 (42%) in the pituitaries of HE vs. LE steers to result in increased expression of *POMC* and *PCSK1*. However, microarray (S3 Table) and RT-PCR (Table 4) results showed no difference in expression level of *NEUROD1* and *TBX19* mRNA in pituitaries of HE vs. LE steers, whereas both *POMC* and *PCSK1*were down-regulated.

Although evidence shows that miRNAs can also up-regulate gene expression [53], an alternative explanation to the above inconsistencies could be due to the stringency level of P-values that was applied to the microarray analysis. Any given gene is usually regulated by numerous miRNAs, and the complements of these miRNAs decide the fate of the transcription of the gene. Thus, a stringent significant cutoff criterion (P < 0.001) could filter out potential miRNAs targeting genes of interest. For example, one striking finding listed in Table 5 is that no DEMs were identified that targeted *DRD2*, *PRL*, or *POMC*. However, when the P-value was relaxed to 0.05, then multiple miRNAs predicted to target *DRD2* (miR-141, miR-214, miR-584, miR-631, miR-2316, miR-2350, miR-2373, miR-2382, miR-2418, miR-2464) were identified. Additionally, miR-2335 and miR-2399 (predicted to target both *VIP* and its transcription factor NURR1 [54] (encoded by *NR4A2*)) also became candidate regulators of *PRL* expression. Collectively, the evidence suggested that altered expression of miRNAs might have affected mRNA abundance by affecting both pre- and post-transcription events of genes regulating prolactin and POMC/ACTH pathways.

This experiment is part of a comprehensive study to understand the whole body and tissue-specific effects of ergot alkaloid consumption in cattle [5, 6, 55]. The unique pituitary-specific findings of this study are an important contribution to our understanding of how ergot alkaloids exert their deleterious effects on cattle production. In summary, the findings indicate that anterior pituitary functions were globally impaired in steers consuming high-toxic endophyte-infected tall fescue. In addition to inhibiting the abilities to synthesize and secrete prolactin (a function of lactotrophs), ACTH synthesis capacity (a function of corticotrophs) might have been reduced. Canonical pathway analysis also indicated that growth hormone signaling and GnRH signaling were altered in HE vs. LE steers (Table 3). A larger implication of this research may be that it allows for selective breeding for genotypes with a higher resistance to endophyte toxicosis, because the specific genes and networks of genes have now been identified that are susceptible to ergot alkaloids contained in endophyte-infected tall fescue. Likewise, with the identification of putative ergot alkaloid sensitive mechanisms within the pituitary gland, this new knowledge may help to develop dietary treatments that ameliorate the effects of ergot alkaloid ingestion [7].

## Supporting information

S1 Fig The sequences of the real-time RT-PCR products (5’ to 3’ orientation). Within a sequence, underlined nucleotides indicate the forward and reverse primer positions.
(DOCX)

S2 Fig Principle component analysis of microarray transcriptome analysis of 16 pituitary samples from steers grazing high- (HE, n = 8, red dots) or low- (LE, n = 8, blue dots) endophyte-infected forages. The red and blue dots represent linear combinations of the relative expression data, including expression values and variances, of the 26,675 gene transcripts in each Bovine GeneChip.
(DOCX)

S3 Fig Hierarchical cluster analysis of the 542 “focus” genes selected as differentially expressed (ANOVA P-values of < 0.001 and false discovery rates of ≤ 5%) by the pituitary of steers grazing high- (HE, n = 8) vs. low- (LE, n = 8) endophyte-infected forages. As indicated by the legend color box, white color in the middle represents the mean value, 0; red color represents gene expression levels above the mean expression; and blue color denotes expression below the mean. The intensity of the color reflects the relative intensity of the fold change.
(DOCX)

S1 Table Primer sets used for quantitative real-time RT-PCR analysis of the selected differentially expressed genes and reference genes. (DOCX)

S2 Table List of differentially expressed pituitary genes (P < 0.001, 542 genes) collected from steers grazing high- (HE, n = 8) or low- (LE, n = 8) endophyte-infected forages. (DOCX)

S3 Table List of selected genes involved in prolactin or POMC/ACTH expression expressed by pituitaries collected from steers grazing high- (HE, n = 8) or low- (LE, n = 8) endophyte-infected forages. (DOCX)
