# Supplementary material for: Pituitary genomic expression profiles of steers are altered by grazing of high vs. low endophyte-infected tall fescue forages
Source: PLoS One. 2017 Sep 13;12(9):e0184612. doi: 10.1371/journal.pone.0184612 (PMC5597216; doi:10.1371/journal.pone.0184612)
Supplement: S1 Fig — Within a sequence, underlined nucleotides indicate the forward and reverse primer positions. (DOCX) [file pone.0184612.s001.docx]

**Supplemental Figure S1.** The sequences of the real-time RT-PCR products (5’ to 3’ orientation). Within a sequence, underlined nucleotides indicate the forward and reverse primer positions.

***ACTB*:**

GAGCGGGAAATCGTCCGTGACATCAAGGAGAAGCTCTGCTACGTGGCCCTGGACTTCGAGCAGGAGATGGCCACCGCGGCCTCCAGCTCCTCCCTGGAGAAGAGCTACGAGCTTCCTGACGGGCAGGTCATCACCATCGGCAATGAGCGGTTCCGCTGCCCTGAGGCTCTCTTCCAGCCTTCCTTCCTGGGCATGGAATCCTGCGGCATTCACGAAACTACCTTCAATTCCATCATGAAGTGTGACGTCGACATCCGCAAGGACCTCTACACCAACAC

***PPIA*:**

GGCAAGTCCAATTATGGCGAGAAATTTGATGATGAGAATTTCATTTTGAAGCATACAGGTCCTGGCATCTTGTCCATGGCAAATGCTGGCCCCAACACAAATGGTTCCCAGTTTTTCATTTGCACTGCCAAGACTGAGTGGTTGGATGGCAAGCACGTGGTCTTTGGCAAGGTGAAAGAGGGCATGAATATTGTGGAAGCCATGGAGCGCTTTGGGTCCAGGAATGGCAAGACCAGCAA

***UBC*:**

TAGGGGTGGGTTAGAGTTCAAGGTTTTTGTTCTACCAGATGTTTTAGTAGTAATCTGGAGGTAAGAAATGTCAAGAAAACATGGCCTTAATTAGAACTGTAGTGGGTGAGTATAAATAAAAAATTTGGAGGTTGTAGTTAGAATTCTCCATATGTACACTCATATGTAGATCTACTTATAAGCTACTGATTTTTAAAAGCACACGTTTGGGAGTTGTGCTTAAGAGTGGGAAAGTTTCTGGAATACCAGCAGGGAGGT

***DRD2*:**

CGACCTTTCTCTGGGGCTTTGGGGCTCTGCGGCTGCGGGGCCAGTATCGAGGCTCGGAGGCCTGGTTTTCACAGGCCATGCCGGAGCTGGTGGTGGGGAGGAGTGGACAGTCACAGCCACCCAGGGCCCACACCTGAGAAGCCAGAGCTCTGGCCACGACCCCAGGCAGTGTCAAGCCTGGGAGACCCGCGTACACCCCAGGTCTGGATGGACCCCAGAGAAGCAGAAGCCCAA

***PRL*:**

AGAACAAGCCCAACAGACCCACCATGAAGTCCTTATGAGCTTGATTCTTGGGTTGCTGCGCTCCTGGAATGACCCTCTGTATCACCTAGTCACCGAGGTGCGGGGTATGAAAGGAGCCCCAGATGCTATCCTATCGAGGGCCATAGAGATTGAGGAAGAAAACAAACGACTTCTGGAAGGCATGGAGATGATATTTGGCCAGGTTATTCCGAGCGCCAAAGAGACTGAGCCCTACCCTGTGTGGTCAGGACT

***s-PRLR*:**

GCCATCCTTTCTGCTGTCATCTGTTTGATTATGGTCTGGGCAGTGGCTTTGAGGGCTATAGCATGGTGACCTGCATCCTCCCACCAGTTCCAGGGCCAAAAATAAAAGGATTTGATGTTCATCTGCTGGAGATATCACAGCCTTCTCGCCT

***l-PRLR*:**

CCATCCTTTCTGCTGTCATCTGTTTGATTATGGTCTGGGCAGTGGCTTTGAAGGGCTATAGCATGGTGACCTGCATCCTCCCACCAGTTCCAGGGCCAAAAATAAAAGGATTTGATGTTCATCTGCTGGAGAAGGG

***POU1F1 (Pit-1)*:**

AAGCAAGAGTGTTGAAGTTTGGTTTGATTTTTCTCTTTGACATGAAAAATAAGTATCTTGTTTCATCACACTATGAAGAAAAGCAAGGCCAGTGAAAGTGTAGAAATAAATTTATTGAGAAGGTAAATAATGAGAGAATAAAATATATAGGGAAAGTTTCTACACAATGTGGCATAGGTGTGAAGTGGTGAAATGATTCTTTTTAATGTATCCAGATTTTTTCCTGCTGTGCTATATACTGTAGTAATTATTCATGAATCATTTTTACAACCTAATATAAGTGTAGCCAGAGCATTCGCACACACCGTTCTTTCTAGTGAATAGCAAGCAATTGCTAGATGAACAATTTAATGTGATAAAAATTATCTACTTATATTAATGTCAAGGCTGGCTAAAGAGCA

***GAL*:**

CACCGGTGAAGGAGAAGAGAGGCTGGACCCTGAACAGCGCTGGGTACCTTCTCGGACCACATGCGCTCGACAGCCACAGGTCATTTCAAGACAAGCATGGCCTCGCCGGCAAGCGGGAACTCGAGCCTGAAGACGAAGCCCGGCCAGGAAGCTTTGACAGACCACTGGCGGAGAACAACGTCGTGCGCACGATAATCGAGTTTCTGACTTTCCTGCATCTCAAAGACGCC

***VIP*:**

CTGGTTCAGCTGTAAGGGCAAGAGAACTCGTGAAGACTGTCGACTCCCAGGACTTCAACACCTGAGACAGCTCTCATAATCTCAACAGAAAGCTCTCAAAGAACACTATTCGGCAAAGTCCTGCAATGGAAACAAGAAGTAAGCCCCAGCTTCTTGTGTTCCTGACGCTGTTCAGCGTGCTCTTCTCCCAGACCTTGGCGTGGCCTCTTTTTGGAGCACCTTCGGCTCTGAGGATGGGGGACAGAATACCATTTGAAGGAGCGAATGAACCTGATCAAGTTTCGTTAAAAGCAGACACTGACATTTTACAAGATGCGCTGGCTGA

***POMC*:**

AGCTTCCCCCTGACAGAGCCTCAGCCTGCCTGGAAGATGCCGAGACTGTGCAGCAGTCGTTCGGGCGCCCTGCTGCTGGCCTTGCTGCTTCAGGCCTCCATGGAAGTGCGTGGTTGGTGCCTGGAGAGCAGCCAGTGTCAGGACCTCACCACGGAAAGTAACCTGCTGGCGTGCATCCGGGCCTGCAAGCCCGACCTCTCCGCCGAGACGCCGGTGTTCCCCGGCAACGGCGACGAGCAGCCGCTGACTGAGAACCCCCGGAAGTACGTCATGGGCCATTTCCGCTGGGACCGCTTCGGCCGTCGGAATGGTAGCAG

***PCSK1*:**

TGATCGTGTGATATGGGCGGAACAACAGTATGAAAAAGAAAGAAGTAAACGTTCAGTTCTAAGAGACTCAGCACTAGATCTCTTCAATGATCCGATGTGGAATCAGCAGTGGTACTTGCAAGATACAAGGATGACTGCAACCCTGCCCAAGCTGGATCTCCATGTGATACCTGTTTGGCAAAAAGGCATCACAGGCAAAGGTGTTGTTATCACTGTATTGGATGATGGCTTGGAGTGGAATCACACAGACATCTATGCCAACTATGATCCGGAGGCC

***GH1:***

CCCAGCAGAAAANCAGACTGGAGCTGCTTCGCATCTCaCTGCTCCTCATCCAGTCGTGGCTTGGGCCCCTGCAGTTCCTCAGCAGAGTCTTCACCAACAGCTTGGTGTTTGGCACCTCGGACCGTGTCTATGAGAAGCTGAAGGACCTGGAGGAAGGCATCCTGGCCCTGATGCGGGAGCTGGAAGATGGCACCCCCCGGGCTGGGCAGATCCTCAAGCAGACCTATGACAAATTTGACACAAACATGCNCAGTGACGA

***TSHB:***

TTTTTGGCCTTGCATGTGGACAAGCAATGTCTTTTTGTATtCcaACTGAGTATATGATGCATGTCGAAAGGAAAGAATGTGCTTACTGCCTAACCATCAACACCACCGTCTGTGCTGGATATTGTATGACACGGGATGTCAACGGCAAGCTGTTTCTTCCCAAATATGCCCTGTCTCAGGATGTCTGTACATACAGAGACTTCATGTACAAGACTGCAGAAATACCAGGATGCCCACGCCTGGTTACTCCT

***TBX19 (Tpit):***

CCATGCCATGCTGGCAACAAAAGTTCGTTGAGTACCTTCCTTGTGcCCTGACCCTTACTCTCAAATGATTCTAGAAGGCTGCCCCTCCTGTTCTGTGGACATAGTGTAAAGCTGGTAAGTGAGCTAAAAATTTACCTAGCCTAAGGCTACTGCATCATAACTTTGTACAGTCCTAAAGGTATGTGTAGCCACAGAGCTTCTGCTTTGAGACCAAGAGAGGGTGTAAGGCCATGGAGTGAAGTTAACAGAAGTAGAGTTAATTACATGTTATAGCACTGAGAATTGAGGAAATGAAATCATGTTGCTCCTTGSTCACAAAC

***NEUROD1:***

GGACAGCTCCCCAGTCTTCCACGTCAAGCCGCCGCCTCACGCCTACAGCGCAGCGCTGGAACCTTTCTTTGAGAGCCCTCTGACTGACTGCACCAGCCCTTCCTTTGACGGACCCCTCAGCCCGCCGCTCAGCATCAATGGCAACTTCTCTTTCAAACACGAACCGTCCGCCGAGTTTGAGAAAAATTATGCCTTtACCATGCACTaTcCTGCAGCGACCCTGGCAGGGGCCCAAAGCCMSGGGATCAGTC

***NR3C1 (Glucocorticoid receptor):***

AAAGAGCAGTGGGAGGACAGCACAATTATCTTTGTGCTGGAAGAAATGATTGTATCATTGATAAAATTCGAAGAAAAAACTGCCCAGCATGCCGCTATAGAAAATGCCTTCAAGCTGGAATGAACCTGGAAGCTCGAAAAACAAAGAAAAAGATAAAAGGAATTCAGCAGGCCACTACGAGGAGTCTCGCANAGAAACATCTGAAAATCCTGCTAACAAAACAATAGTTCCTGCAACATTACCACAACTCCCCGACGCT

***CRHR1:***

NNAGCAAGGNTCACTACCACATCGCTGTCATCATCAACTACCTAGGCCACTGCATCTCCCTGGCGGCCCTCCTGGTGGCCTTTGTCCTCTTTCTGCGGCTCAGGAGCATCCGGTGCCTGAGAAACATCATCCACTGGAACCTCATCTCAGCCTTCATCCTGCGCAATGCCACGTGGTTCGTGGTCCAGCTCACCATGAGCCCCGAAGTCCATCAGAGCAACGTGGGCTGGTGCAGGCTGGTGACAGCCGCCTACAACTACTTCCACGTGACCAACTTCTTCTGGATGTTCGGTGAGGGCTGCTACCTGCACACGGCCATCGTGCTCACGTCTACCACAGACC

***CRHR2:***

GCTGGTTTTGGAGGCTGGGGGCTGCCCCTGCACCCCGAGGGTCCCTACTCCTACTGCAACACGACCTTGGACCAGATCGGGACGTGCTGGCCCCGGAGCTCGGCCGGAGCCCTGGTGGAGAGGCCGTGCCCCGAGTACTTCAACGGTGTCAAGTACAACACGACCCGGAATGCCTACCGAGAGTGCTTGGAGAATGGGACGTGGGCCTCGCGGATCAACTACTCACA
