# Supplementary material for: Pituitary genomic expression profiles of steers are altered by grazing of high vs. low endophyte-infected tall fescue forages
Source: PLoS One. 2017 Sep 13;12(9):e0184612. doi: 10.1371/journal.pone.0184612 (PMC5597216; doi:10.1371/journal.pone.0184612)

**Supplemental Figure S2.** Principle component analysis of microarray transcriptome analysis of 16 pituitary samples from steers grazing high- (HE, n = 8, red dots) or low- (LE, n = 8, blue dots) endophyte-infected forages. The red and blue dots represent linear combinations of the relative expression data, including expression values and variances, of the 26,675 gene transcripts in each Bovine GeneChip.
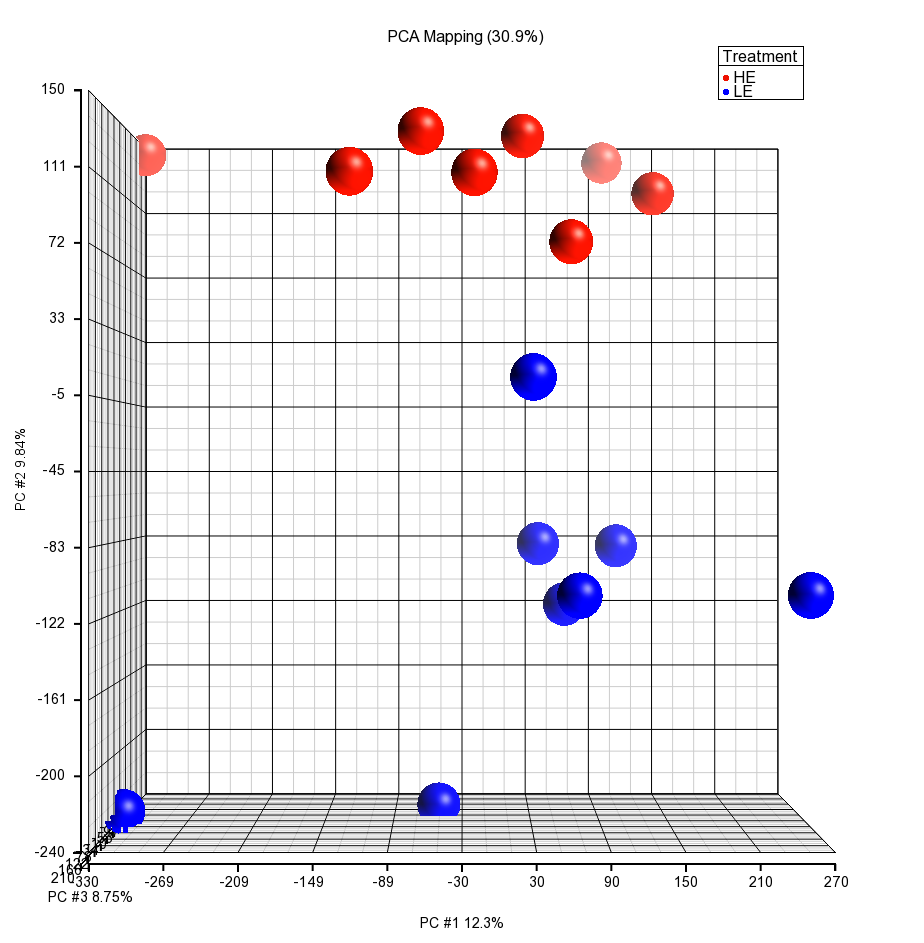

Supplement: S2 Fig — The red and blue dots represent linear combinations of the relative expression data, including expression values and variances, of the 26,675 gene transcripts in each Bovine GeneChip. (DOCX) [file pone.0184612.s002.docx]
