# Supplementary material for: Pituitary genomic expression profiles of steers are altered by grazing of high vs. low endophyte-infected tall fescue forages
Source: PLoS One. 2017 Sep 13;12(9):e0184612. doi: 10.1371/journal.pone.0184612 (PMC5597216; doi:10.1371/journal.pone.0184612)
Supplement: S3 Fig — As indicated by the legend color box, white color in the middle represents the mean value, 0; red color represents gene expression levels above the mean expression; and blue color denotes expression below the mean. The intensity of the color reflects the relative intensity of the fold change. (DOCX) [file pone.0184612.s003.docx]

**Supplemental Figure S3.** Hierarchical cluster analysis of the 542 “focus” genes selected as differentially expressed (ANOVA P-values of < 0.001 and false discovery rates of ≤ 5%) by the pituitary of steers grazing high- (HE, n = 8) vs. low- (LE, n = 8) endophyte-infected forages. As indicated by the legend color box, white color in the middle represents the mean value, 0; red color represents gene expression levels above the mean expression; and blue color denotes expression below the mean. The intensity of the color reflects the relative intensity of the fold change.

**
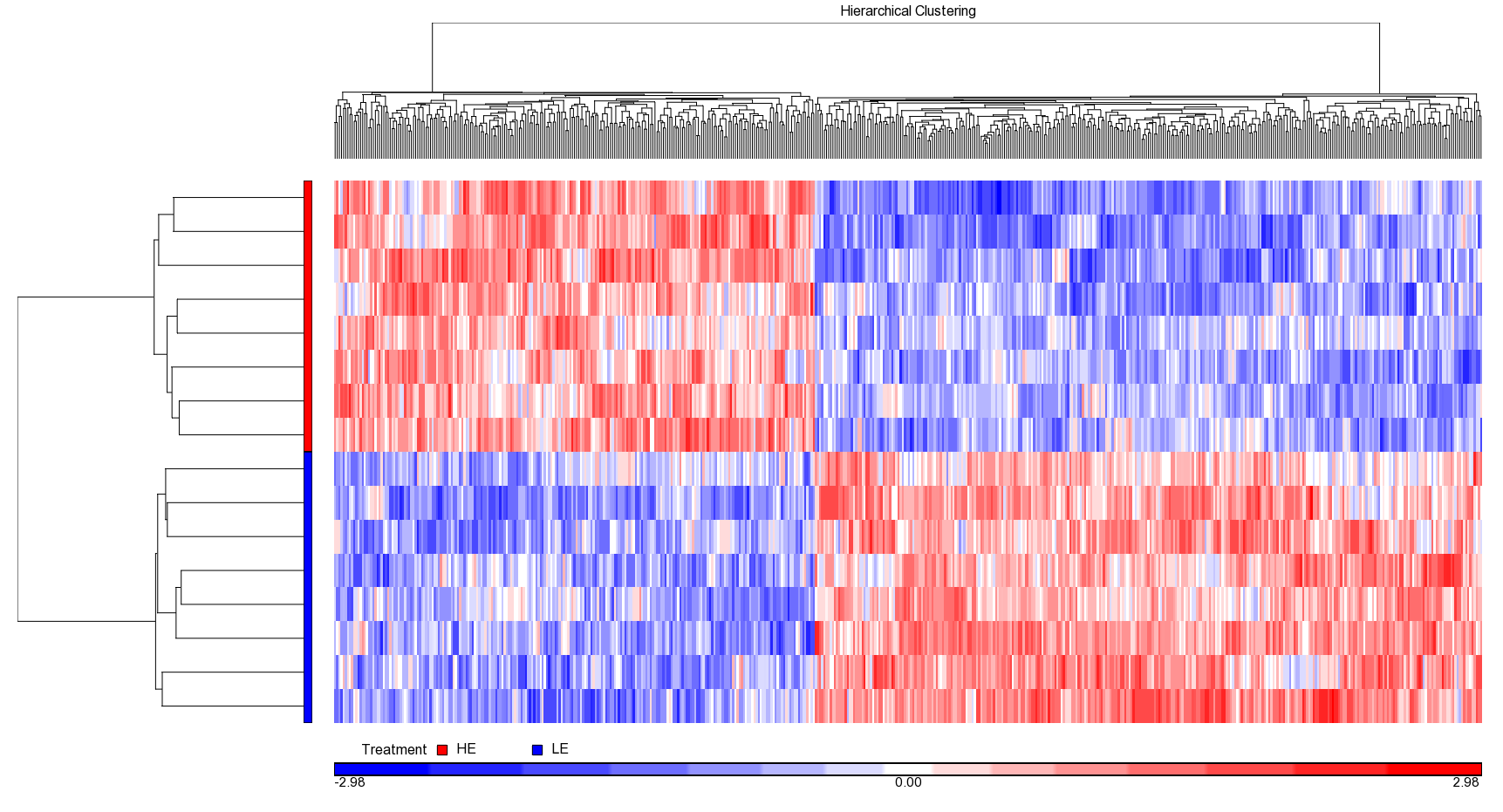
**
