# Supplementary material for: Pituitary genomic expression profiles of steers are altered by grazing of high vs. low endophyte-infected tall fescue forages
Source: PLoS One. 2017 Sep 13;12(9):e0184612. doi: 10.1371/journal.pone.0184612 (PMC5597216; doi:10.1371/journal.pone.0184612)
Supplement: S1 Table — (DOCX) [file pone.0184612.s004.docx]

| **Supplemental Table 1.** Primer sets used for quantitative real-time RT-PCR analysis of the selected differentially expressed genes and reference genes. | | | | | |  |
| --- | --- | --- | --- | --- | --- | --- |
| Gene | Gene Name | Primer & Accession number^1^ | Sequence (5' to 3' direction) | Amplicon length (bp) | Product identity (%)^2^ |  |
| *ACTB* | Actin, beta | *NM_173979.3* | |  |  |  |
|  |  | Forward | GAGCGGGAAATCGTCCGTGAC | 278 | 99 |  |
|  |  | Reverse | GTGTTGGCGTAGAGGTCCTTGC |  |  |  |
| *PPIA* | Peptidylprolyl isomerase A | *NM_178320.2* | |  |  |  |
|  |  | Forward | GGCAAGTCCATCTATGGCGA | 239 | 99 |  |
|  |  | Reverse | TTGCTGGTCTTGCCATTCCT |  |  |  |
| *UBC* | Ubiquitin C | *NM_001206307.1* | |  |  |  |
|  |  | Forward | TAGGGGTGGGTTAGAGTTCAAG | 258 | 100 |  |
|  |  | Reverse | ACCACCTCCCTGCTGGTATT |  |  |  |
| *DRD2* | Dopamine receptor D2 | *NM_174043.2* | |  |  |  |
|  |  | Forward | CGACCTTTCTCTGGGGCTTT | 234 | 100 |  |
|  |  | Reverse | TTGGGCTTCTGCTTCTCTGG |  |  |  |
| *PRL* | Prolactin | *NM_173953.2* | |  |  |  |
|  |  | Forward | AGAACAAGCCCAACAGACCC | 252 | 99 |  |
|  |  | Reverse | AGTCCTGACCACACAGGGTA |  |  |  |
| *s-PRLR* | Short Prolactin receptor | *NM_174155.3* |  |  |  |  |
|  |  | Forward | GCCATCCTTTCTGCTGTCAT | 151 | 99 |  |
|  |  | Reverse | AAGGCGAGAAGGCTGTGATA |  |  |  |
| *l-PRLR* | Long Prolactin receptor | *NM_001039726.2* | |  |  |  |
|  |  | Forward | GCCATCCTTTCTGCTGTCAT | 136 | 100 |  |
|  |  | Reverse | CCCTTCTCCAGCAGATGAAC |  |  |  |
| *POU1F1* | POU class 1 homeobox 1 | *AH012495.1* | |  |  |  |
|  |  | Forward | AAGCAAGAGGTTTGAAGTTTGGT | 401 | 99 |  |
|  |  | Reverse | TGCTCTTTAGCCAGCCTTGA |  |  |  |
| *GAL* | Galanin/GMAP prepropeptide | *NM_173914.2* |  |  |  |  |
|  |  | Forward | CACCGGTGAAGGAGAAGAGAG | 230 | 100 |  |
|  |  | Reverse | GGCGTCTTTGAGATGCAGGAA |  |  |  |
| *VIP* | Vasoactive intestinal peptide | *NM_173970.3* |  |  |  |  |
|  |  | Forward | CTGGTTCAGCTGTAAGGGCA | 325 | 100 |  |
|  |  | Reverse | TCAGCCAGCGCATCTTGTAA |  |  |  |
| *POMC* | Proopiomelanocortin | *NM_174151.1* |  |  |  |  |
|  |  | Forward | AGCTTCCCCGTGACAGAGC | 317 | 99 |  |
|  |  | Reverse | CTGCTACCATTCCGACGGC |  |  |  |
| *PCSK1* | Proprotein convertase sutilisin/kexin type 1 | *NM_174412.2* |  |  |  |  |
|  |  | Forward | TGATCGTGTGATATGGGCGG | 277 | 100 |  |
|  |  | Reverse | GGCCTCCGGATCATAGTTGG |  |  |  |
| *GH1* | Growth Hormone 1 | *NM_180996.1* |  |  |  |  |
|  |  | Forward | CCCAGCAGAAATCAGACTTGGA | 260 | 98 |  |
|  |  | Reverse | GTCGTCACTGCGCATGTTTG |  |  |  |
| *TSHB* | Thyroid stimulating hormone beta | *NM_174205.1* |  |  |  |  |
|  |  | Forward | TTTTGGCCTTGCATGTGGAC | 251 | 99 |  |
|  |  | Reverse | AAGGAGTAACATGGCGTGGG |  |  |  |
| *TBX19* | T-Box 19 | *NM_001075663.1* |  |  |  |  |
|  |  | Forward | CCATGCCATGCTTAGGCAAC | 325 | 98 |  |
|  |  | Reverse | AGGTTTCTGAGCCAAGGAGC |  |  |  |
| *NeuroD1* | Neuronal differentiation 1 | *NM_001103288.1* |  |  |  |  |
|  |  | Forward | GGACAGCTCCCATGTCTTCC | 251 | 98 |  |
|  |  | Reverse | AGACTGATCCGTGGCTTTGG |  |  |  |
| *NR3C1* | Nuclear receptor subfamily 3 group C member 1 | *NM_001206634.1* |  |  |  |  |
|  |  | Forward | AAAGAGCAGTGGAAGGACAGC | 261 | 98 |  |
|  |  | Reverse | CCAGCGTAGGTGTGAGTTGT |  |  |  |
| *CRHR1* | Corticotropin releasing hormone receptor 1 | *NM_174287.1* |  |  |  |  |
|  |  | Forward | AGAGCAAGGTGCACTACCAC | 342 | 99 |  |
|  |  | Reverse | GGTCTGTGGAGTACGTGAGC |  |  |  |
| *CRHR2* | Corticotropin releasing hormone receptor 2 | *NM_001192545.1* |  |  |  |  |
|  |  | Forward | GCTGGTTTTGGACGGCTG | 228 | 99 |  |
|  |  | Reverse | TGTGAGTAGTTGATCCGCGA |  |  |  |

^1^The contents in the parentheses associated with each gene symbol are the accession numbers of the sequences retrieved from the NCBI RefSeq database and used as templates for designing primers and probes.

^2^All the real-time RT-PCR products were validated by sequencing. The identity values (%) presented are the base-pair ratios between the number of identical base pairs and the total amplicon length.
