# Supplementary material for: Pituitary genomic expression profiles of steers are altered by grazing of high vs. low endophyte-infected tall fescue forages
Source: PLoS One. 2017 Sep 13;12(9):e0184612. doi: 10.1371/journal.pone.0184612 (PMC5597216; doi:10.1371/journal.pone.0184612)
Supplement: S2 Table — (DOCX) [file pone.0184612.s005.docx]

**Supplemental Table 2.** List of differentially expressed pituitary genes (P < 0.001, 542 genes) collected from steers grazing high- (HE, n = 8) or low- (LE, n = 8) endophyte-infected forages.

| Transcript ID | Gene Symbol | Gene assignment | p-value | False discovery Rate | Ratio(HE vs.LE) | Fold-Change(HE vs. LE) |
| --- | --- | --- | --- | --- | --- | --- |
| 12876894 | GPX3 | glutathione peroxidase 3 (plasma) | 8.90E-10 | 2.37E-05 | 0.479140613 | -2.08707 |
| 12851243 | ASB4 | ankyrin repeat and SOCS box containing 4 | 1.73E-08 | 0.000230196 | 0.526931467 | -1.89778 |
| 12898500 | EPHA7 | EPH receptor A7 | 2.67E-08 | 0.000237549 | 1.797668783 | 1.79767 |
| 12835339 | COPA | coatomer protein complex, subunit alpha | 5.72E-08 | 0.000332512 | 0.906996572 | -1.10254 |
| 12798282 | CHL1 | cell adhesion molecule with homology to L1CAM (close homolog of | 6.23E-08 | 0.000332512 | 0.532076215 | -1.87943 |
| 12871398 | PLA2G12A | phospholipase A2, group XIIA | 9.95E-08 | 0.000387737 | 0.761666832 | -1.31291 |
| 12776100 | CPS1 | carbamoyl-phosphate synthase 1, mitochondrial | 1.02E-07 | 0.000387737 | 0.425693135 | -2.34911 |
| 12721755 | MMP16 | matrix metallopeptidase 16 (membrane-inserted) | 2.29E-07 | 0.000764464 | 1.30842271 | 1.30842 |
| 12902267 | BHLHB9 | basic helix-loop-helix domain containing, class B, 9 | 4.95E-07 | 0.00146659 | 0.712230421 | -1.40404 |
| 12730735 | DRD2 | dopamine receptor D2 | 5.55E-07 | 0.00148011 | 0.569216758 | -1.7568 |
| 12737221 | FAM163A | family with sequence similarity 163, member A | 8.61E-07 | 0.00192236 | 0.532013939 | -1.87965 |
| 12780430 | PTPRN | protein tyrosine phosphatase, receptor type, N | 9.71E-07 | 0.00192236 | 0.776578396 | -1.2877 |
| 12866683 | SLC41A2 | solute carrier family 41, member 2 | 1.12E-06 | 0.00192236 | 0.753801042 | -1.32661 |
| 12722069 | CDH17 | cadherin 17, LI cadherin (liver-intestine) | 1.15E-06 | 0.00192236 | 0.309513198 | -3.23088 |
| 12826450 | UNC5B | unc-5 homolog B (C. elegans) | 1.15E-06 | 0.00192236 | 0.617078258 | -1.62054 |
| 12767970 | NSF | N-ethylmaleimide-sensitive factor | 1.20E-06 | 0.00192236 | 0.856927401 | -1.16696 |
| 12836423 | DIRAS3 | DIRAS family, GTP-binding RAS-like 3 | 1.26E-06 | 0.00192236 | 0.731042247 | -1.36791 |
| 12729629 | REXO2 | REX2, RNA exonuclease 2 homolog (S. cerevisiae) | 1.30E-06 | 0.00192236 | 0.638740914 | -1.56558 |
| 12890848 | RORB | RAR-related orphan receptor B | 1.57E-06 | 0.00209906 | 1.373516945 | 1.37352 |
| 12679305 | LNP1 | leukemia NUP98 fusion partner 1 | 1.62E-06 | 0.00209906 | 0.838609585 | -1.19245 |
| 12866960 | PLEKHA5 | pleckstrin homology domain containing A5 | 1.65E-06 | 0.00209906 | 1.257116853 | 1.25712 |
| 12765517 | RPRML | reprimo-like | 1.81E-06 | 0.00219319 | 0.772260406 | -1.2949 |
| 12821658 | ABLIM1 | actin binding LIM protein 1 | 2.06E-06 | 0.00230454 | 1.308681268 | 1.30868 |
| 12909850 | SLITRK2 | SLIT and NTRK-like family, member 2 | 2.07E-06 | 0.00230454 | 1.349001537 | 1.349 |
| 12876760 | PAM | peptidylglycine alpha-amidating monooxygenase | 2.21E-06 | 0.0023559 | 0.51613436 | -1.93748 |
| 12706637 | FAM98A | family with sequence similarity 98, member A | 2.85E-06 | 0.00292358 | 0.814730324 | -1.2274 |
| 12731709 | ZW10 | ZW10, kinetochore associated, homolog (Drosophila) | 3.33E-06 | 0.00326731 | 0.82611174 | -1.21049 |
| 12722190 | MRPL15 | mitochondrial ribosomal protein L15 | 3.46E-06 | 0.00326731 | 0.746246381 | -1.34004 |
| 12883237 | PCSK1 | proprotein convertase subtilisin | 3.55E-06 | 0.00326731 | 0.580548154 | -1.72251 |
| 12901396 | PKIB | protein kinase (cAMP-dependent, catalytic) inhibitor beta | 3.92E-06 | 0.00348198 | 0.710292854 | -1.40787 |
| 12844085 | PEA15 | phosphoprotein enriched in astrocytes 15 | 4.07E-06 | 0.00350175 | 1.182574062 | 1.18257 |
| 12725728 | EFR3A | EFR3 homolog A (S. cerevisiae) | 4.33E-06 | 0.00350945 | 0.759999696 | -1.31579 |
| 12796976 | SLC6A11 | solute carrier family 6 (neurotransmitter transporter, GABA) | 4.34E-06 | 0.00350945 | 1.353280691 | 1.35328 |
| 12681884 | PLSCR4 | phospholipid scramblase 4 | 4.85E-06 | 0.00380365 | 1.340186152 | 1.34019 |
| 12698445 | ODC1 | ornithine decarboxylase 1 | 5.00E-06 | 0.00381304 | 0.720964939 | -1.38703 |
| 12738110 | APOBEC4 | apolipoprotein B mRNA editing enzyme, catalytic polypeptide- | 5.77E-06 | 0.00411905 | 0.58433742 | -1.71134 |
| 12690836 | RORA | RAR-related orphan receptor A | 5.81E-06 | 0.00411905 | 1.179369061 | 1.17937 |
| 12804415 | PRL | prolactin | 6.12E-06 | 0.00411905 | 0.814617496 | -1.22757 |
| 12884669 | DPYSL3 | dihydropyrimidinase-like 3 | 6.12E-06 | 0.00411905 | 0.623526918 | -1.60378 |
| 12820770 | TCF7L2 | transcription factor 7-like 2 (T-cell specific, HMG-box | 6.28E-06 | 0.00411905 | 1.215108169 | 1.21511 |
| 12681044 | ADPRH | ADP-ribosylarginine hydrolase | 6.55E-06 | 0.00411905 | 0.79231141 | -1.26213 |
| 12816646 | WBSCR22 | Williams Beuren syndrome chromosome region 22 | 6.57E-06 | 0.00411905 | 0.832625602 | -1.20102 |
| 12909727 | GPR101 | G protein-coupled receptor 101 | 6.64E-06 | 0.00411905 | 0.372790749 | -2.68247 |
| 12684305 | ITSN1 | intersectin 1 (SH3 domain protein) | 7.11E-06 | 0.00423958 | 1.169628947 | 1.16963 |
| 12694513 | HEATR4 | HEAT repeat containing 4 | 7.15E-06 | 0.00423958 | 1.248296076 | 1.2483 |
| 12854099 | ASNS | asparagine synthetase (glutamine-hydrolyzing) | 8.47E-06 | 0.00486962 | 0.823092688 | -1.21493 |
| 12835795 | CD247 | CD247 molecule | 8.58E-06 | 0.00486962 | 1.181139563 | 1.18114 |
| 12760666 | CLTC | clathrin, heavy chain (Hc) | 8.86E-06 | 0.00492327 | 0.913567389 | -1.09461 |
| 12891421 | NFIB | nuclear factor I | 9.41E-06 | 0.00512338 | 1.240334692 | 1.24033 |
| 12796050 | EXOG | endo | 9.64E-06 | 0.00514056 | 0.828665186 | -1.20676 |
| 12744882 | CIT | citron (rho-interacting, serine | 9.90E-06 | 0.00518005 | 0.789914373 | -1.26596 |
| 12728901 | SORL1 | sortilin-related receptor, L(DLR class) A repeats contai | 1.03E-05 | 0.00529555 | 1.242348685 | 1.24235 |
| 12863173 | IQSEC3 | IQ motif and Sec7 domain 3 | 1.09E-05 | 0.00549229 | 0.695574753 | -1.43766 |
| 12754444 | CPNE2 | copine II | 1.13E-05 | 0.00557045 | 0.668847108 | -1.49511 |
| 12772434 | ENO3 | enolase 3 (beta, muscle) | 1.19E-05 | 0.0057655 | 0.652213613 | -1.53324 |
| 12849880 | RINT1 | RAD50 interactor 1 | 1.22E-05 | 0.00577337 | 0.84404568 | -1.18477 |
| 12845993 | CADM3 | cell adhesion molecule 3 | 1.24E-05 | 0.00577337 | 1.336918056 | 1.33692 |
| 12862258 | RNF41 | ring finger protein 41 | 1.26E-05 | 0.00577337 | 0.848874816 | -1.17803 |
| 12760918 | TIMP2 | TIMP metallopeptidase inhibitor 2 | 1.36E-05 | 0.00592615 | 0.828919338 | -1.20639 |
| 12890327 | LPPR1 | lipid phosphate phosphatase-related protein type 1 | 1.36E-05 | 0.00592615 | 0.703650539 | -1.42116 |
| 12721605 | FABP5 | fatty acid binding protein 5 (psoriasis-associated) | 1.38E-05 | 0.00592615 | 0.630250777 | -1.58667 |
| 12772567 | CDR2L | cerebellar degeneration-related protein 2-like | 1.39E-05 | 0.00592615 | 0.610884744 | -1.63697 |
| 12818366 | VGF | VGF nerve growth factor inducible | 1.40E-05 | 0.00592615 | 0.688477638 | -1.45248 |
| 12909004 | KLHL13 | kelch-like 13 (Drosophila) | 1.42E-05 | 0.00592615 | 0.647261759 | -1.54497 |
| 12681831 | BCL6 | B-cell CLL | 1.45E-05 | 0.00594922 | 1.390644578 | 1.39064 |
| 12887101 | CLINT1 | clathrin interactor 1 | 1.51E-05 | 0.00611067 | 0.902535221 | -1.10799 |
| 12822767 | GFRA1 | GDNF family receptor alpha 1 | 1.57E-05 | 0.00625298 | 1.370279729 | 1.37028 |
| 12825420 | ACSL1 | acyl-CoA synthetase long-chain family member 1 | 1.60E-05 | 0.00628789 | 1.263664957 | 1.26366 |
| 12744620 | KSR2 | kinase suppressor of ras 2 | 1.64E-05 | 0.00632783 | 0.776831769 | -1.28728 |
| 12734598 | PRELP | proline | 1.68E-05 | 0.00640273 | 1.297572242 | 1.29757 |
| 12812405 | TPST1 | tyrosylprotein sulfotransferase 1 | 1.70E-05 | 0.00640273 | 0.755635149 | -1.32339 |
| 12780755 | RCAN3 | RCAN family member 3 | 1.78E-05 | 0.00654189 | 0.616663481 | -1.62163 |
| 12830994 | LRRC10B | leucine rich repeat containing 10B | 1.79E-05 | 0.00654189 | 0.774521346 | -1.29112 |
| 12717875 | LOC100335867 | uncharacterized LOC100335867 | 1.87E-05 | 0.00672986 | 0.702864171 | -1.42275 |
| 12852359 | CLEC2L | C-type lectin domain family 2, member L | 1.94E-05 | 0.00685045 | 0.511872892 | -1.95361 |
| 12851732 | COBL | cordon-bleu homolog (mouse) | 1.95E-05 | 0.00685045 | 1.226814735 | 1.22681 |
| 12800654 | FAM19A1 | family with sequence similarity 19 (chemokine (C-C motif)-li | 2.03E-05 | 0.00698948 | 0.491956511 | -2.0327 |
| 12791580 | SERPINA1 | serpin peptidase inhibitor, clade A (alpha-1 antiproteinase, a | 2.04E-05 | 0.00698948 | 0.37702557 | -2.65234 |
| 12914851 |  |  | 2.18E-05 | 0.00725635 | 0.338304149 | -2.95592 |
| 12894663 | KANK1 | KN motif and ankyrin repeat domains 1 | 2.22E-05 | 0.00725635 | 1.228496701 | 1.2285 |
| 12880336 | RHOBTB3 | Rho-related BTB domain containing 3 | 2.22E-05 | 0.00725635 | 0.778446377 | -1.28461 |
| 12690776 | C10H15orf61 | chromosome 10 open reading frame, human C15orf61 | 2.25E-05 | 0.00725635 | 0.822206144 | -1.21624 |
| 12843689 | IL6R | interleukin 6 receptor | 2.26E-05 | 0.00725635 | 1.272669424 | 1.27267 |
| 12785900 | SERF1A | small EDRK-rich factor 1A (telomeric) | 2.29E-05 | 0.00725635 | 0.793361154 | -1.26046 |
| 12900382 | IPCEF1 | interaction protein for cytohesin exchange factors 1 | 2.32E-05 | 0.00729635 | 0.665734638 | -1.5021 |
| 12749473 | CLEC3A | C-type lectin domain family 3, member A | 2.40E-05 | 0.00744792 | 0.495554873 | -2.01794 |
| 12904860 | TMEM35 | transmembrane protein 35 | 2.56E-05 | 0.00786195 | 0.773108204 | -1.29348 |
| 12786942 | SDHA | succinate dehydrogenase complex, subunit A, flavoprotein (Fp) | 2.60E-05 | 0.00788154 | 0.90721874 | -1.10227 |
| 12901161 | FAM46A | family with sequence similarity 46, member A | 2.69E-05 | 0.00802629 | 0.648193162 | -1.54275 |
| 12718471 | PCMTD2 | protein-L-isoaspartate (D-aspartate) O-methyltransferase doma | 2.71E-05 | 0.00802629 | 1.159134034 | 1.15913 |
| 12743540 | GOLGA3 | golgin A3 | 2.92E-05 | 0.00856137 | 0.858288059 | -1.16511 |
| 12914547 |  |  | 3.08E-05 | 0.00876601 | 0.788867502 | -1.26764 |
| 12694561 | SIX1 | SIX homeobox 1 | 3.11E-05 | 0.00876601 | 0.862529973 | -1.15938 |
| 12845091 | GIPC2 | GIPC PDZ domain containing family, member 2 | 3.11E-05 | 0.00876601 | 0.749198358 | -1.33476 |
| 12798897 | SPCS1 | signal peptidase complex subunit 1 homolog (S. cerevisiae) | 3.12E-05 | 0.00876601 | 0.82606397 | -1.21056 |
| 12705554 | KCNIP3 | Kv channel interacting protein 3, calsenilin | 3.19E-05 | 0.00885044 | 0.337708915 | -2.96113 |
| 12900949 | CLVS2 | clavesin 2 | 3.27E-05 | 0.00898575 | 0.460095884 | -2.17346 |
| 12865096 | CPT1B | carnitine palmitoyltransferase 1B (muscle) | 3.42E-05 | 0.00924483 | 0.766530224 | -1.30458 |
| 12730077 | ART5 | ADP-ribosyltransferase 5 | 3.43E-05 | 0.00924483 | 0.702656745 | -1.42317 |
| 12891381 | RFX3 | regulatory factor X, 3 (influences HLA class II expression) | 3.65E-05 | 0.00967033 | 1.154782416 | 1.15478 |
| 12718040 | BLCAP | bladder cancer associated protein | 3.66E-05 | 0.00967033 | 1.149770218 | 1.14977 |
| 12885917 | ZNF300 | zinc finger protein 300 | 3.75E-05 | 0.00979751 | 0.591800019 | -1.68976 |
| 12724574 | DENND3 | DENN | 3.80E-05 | 0.00985362 | 0.761162447 | -1.31378 |
| 12807840 | LOC510913 | nose resistant to fluoxetine protein 6-like | 3.97E-05 | 0.0101555 | 0.378382264 | -2.64283 |
| 12722987 | AZIN1 | antizyme inhibitor 1 | 4.01E-05 | 0.0101555 | 0.893367639 | -1.11936 |
| 12824358 | TNKS | tankyrase, TRF1-interacting ankyrin-related ADP-ribose polymera | 4.04E-05 | 0.0101555 | 0.860067085 | -1.1627 |
| 12772732 | PRKAR1A | protein kinase, cAMP-dependent, regulatory, type I, alpha (t | 4.26E-05 | 0.0105979 | 0.91503866 | -1.09285 |
| 12786113 | FGF18 | fibroblast growth factor 18 | 4.33E-05 | 0.0105979 | 0.788562687 | -1.26813 |
| 12815257 | STX1B | syntaxin 1B | 4.33E-05 | 0.0105979 | 1.19426086 | 1.19426 |
| 12782974 | MFSD6 | major facilitator superfamily domain containing 6 | 4.39E-05 | 0.0106441 | 0.862009517 | -1.16008 |
| 12703883 | GFPT1 | glutamine--fructose-6-phosphate transaminase 1 | 4.43E-05 | 0.0106441 | 0.882378893 | -1.1333 |
| 12808014 | SLC39A6 | solute carrier family 39 (zinc transporter), member 6 | 4.48E-05 | 0.0106789 | 0.83467577 | -1.19807 |
| 12709784 | GPC5 | glypican 5 | 4.72E-05 | 0.0110629 | 0.715727394 | -1.39718 |
| 12727699 | CREB3L1 | cAMP responsive element binding protein 3-like 1 | 4.74E-05 | 0.0110629 | 0.674427074 | -1.48274 |
| 12681063 | GNB4 | guanine nucleotide binding protein (G protein), beta polypeptid | 4.77E-05 | 0.0110629 | 0.700461604 | -1.42763 |
| 12733712 | BDNF | brain-derived neurotrophic factor | 4.81E-05 | 0.0110629 | 0.659565346 | -1.51615 |
| 12786160 | OXCT1 | 3-oxoacid CoA transferase 1 | 4.89E-05 | 0.0111294 | 0.823512942 | -1.21431 |
| 12867761 | COQ10A | coenzyme Q10 homolog A (S. cerevisiae) | 4.94E-05 | 0.0111294 | 0.879855704 | -1.13655 |
| 12706846 | PREPL | prolyl endopeptidase-like | 4.98E-05 | 0.0111294 | 0.857581449 | -1.16607 |
| 12816678 | TRAP1 | TNF receptor-associated protein 1 | 5.01E-05 | 0.0111294 | 0.832729604 | -1.20087 |
| 12771103 | TRPV3 | transient receptor potential cation channel, subfamily V, memb | 6.00E-05 | 0.0132377 | 0.691620328 | -1.44588 |
| 12719676 | SLC17A9 | solute carrier family 17, member 9 | 6.24E-05 | 0.0136424 | 0.676823532 | -1.47749 |
| 12767584 | MAP2K6 | mitogen-activated protein kinase kinase 6 | 6.36E-05 | 0.0137836 | 1.493379847 | 1.49338 |
| 12887812 | SHROOM1 | shroom family member 1 | 6.41E-05 | 0.0137836 | 1.109131927 | 1.10913 |
| 12791646 | WARS | tryptophanyl-tRNA synthetase | 6.57E-05 | 0.0140166 | 0.859195106 | -1.16388 |
| 12706360 | SURF4 | surfeit 4 | 6.81E-05 | 0.0143244 | 0.867611206 | -1.15259 |
| 12707532 | COX7A2L | cytochrome c oxidase subunit VIIa polypeptide 2 like | 6.88E-05 | 0.0143244 | 0.798607229 | -1.25218 |
| 12861735 | C5H12orf23 | chromosome 5 open reading frame, human C12orf23 | 6.89E-05 | 0.0143244 | 0.894958698 | -1.11737 |
| 12677247 |  |  | 6.93E-05 | 0.0143244 | 1.748212016 | 1.74821 |
| 12844481 | POU2F1 | POU class 2 homeobox 1 | 7.08E-05 | 0.0144902 | 1.152076387 | 1.15208 |
| 12737098 | B3GALT2 | UDP-Gal:betaGlcNAc beta 1,3-galactosyltransferase, polypepti | 7.16E-05 | 0.0144902 | 0.626550713 | -1.59604 |
| 12758081 | MYLK3 | myosin light chain kinase 3 | 7.18E-05 | 0.0144902 | 0.704002253 | -1.42045 |
| 12765456 | TOM1L2 | target of myb1-like 2 (chicken) | 7.22E-05 | 0.0144902 | 0.841078262 | -1.18895 |
| 12690792 | GNB5 | guanine nucleotide binding protein (G protein), beta 5 | 7.90E-05 | 0.0157305 | 0.901989789 | -1.10866 |
| 12898520 | SASH1 | SAM and SH3 domain containing 1 | 7.98E-05 | 0.0157582 | 1.279225506 | 1.27923 |
| 12741946 | TCN2 | transcobalamin II | 8.15E-05 | 0.0159752 | 0.818618663 | -1.22157 |
| 12904911 | HNRNPH2 | heterogeneous nuclear ribonucleoprotein H2 (H') | 8.20E-05 | 0.0159752 | 0.831213738 | -1.20306 |
| 12713066 | SLITRK6 | SLIT and NTRK-like family, member 6 | 8.42E-05 | 0.016277 | 0.526853735 | -1.89806 |
| 12848963 | GNGT1 | guanine nucleotide binding protein (G protein), gamma transducing | 8.57E-05 | 0.0163351 | 0.471211343 | -2.12219 |
| 12801704 | LOC511316 | MEF2B neighbor pseudogene | 8.58E-05 | 0.0163351 | 0.82234137 | -1.21604 |
| 12780398 | TNS1 | tensin 1 | 8.71E-05 | 0.0163351 | 1.279664216 | 1.27966 |
| 12831896 | NRGN | neurogranin (protein kinase C substrate, RC3) | 8.72E-05 | 0.0163351 | 1.663730183 | 1.66373 |
| 12830772 | SCYL1 | SCY1-like 1 (S. cerevisiae) | 8.79E-05 | 0.0163351 | 0.873438728 | -1.1449 |
| 12785083 | PRLR | prolactin receptor | 8.86E-05 | 0.0163351 | 0.762951095 | -1.3107 |
| 12771233 | LRRC48 | leucine rich repeat containing 48 | 8.88E-05 | 0.0163351 | 0.798103706 | -1.25297 |
| 12778650 | FIGN | fidgetin | 9.04E-05 | 0.0165243 | 1.271622349 | 1.27162 |
| 12693799 | DIO2 | deiodinase, iodothyronine, type II | 9.17E-05 | 0.0166237 | 0.592364423 | -1.68815 |
| 12708635 | AFF3 | AF4 | 9.23E-05 | 0.0166237 | 1.181992579 | 1.18199 |
| 12716197 | TM9SF4 | transmembrane 9 superfamily protein member 4 | 9.29E-05 | 0.0166237 | 0.880211955 | -1.13609 |
| 12822603 | NRAP | nebulin-related anchoring protein | 9.37E-05 | 0.0166607 | 0.664721249 | -1.50439 |
| 12844104 | FAM19A3 | family with sequence similarity 19 (chemokine (C-C motif)-li | 9.43E-05 | 0.0166607 | 0.766101539 | -1.30531 |
| 12696210 | SLC27A2 | solute carrier family 27 (fatty acid transporter), member 2 | 9.63E-05 | 0.0167793 | 0.653628948 | -1.52992 |
| 12808788 | SETBP1 | SET binding protein 1 | 9.69E-05 | 0.0167793 | 1.142543759 | 1.14254 |
| 12891005 | PTPRD | protein tyrosine phosphatase, receptor type, D | 9.70E-05 | 0.0167793 | 1.365526998 | 1.36553 |
| 12855122 | TES | testis derived transcript (3 LIM domains) | 9.75E-05 | 0.0167793 | 0.898303105 | -1.11321 |
| 12808491 | ZNF521 | zinc finger protein 521 | 9.94E-05 | 0.0170016 | 1.332127314 | 1.33213 |
| 12687743 | NEO1 | neogenin 1 | 0.00010109 | 0.0171237 | 1.181993977 | 1.18199 |
| 12886016 | MFSD12 | major facilitator superfamily domain containing 12 | 0.000101426 | 0.0171237 | 0.867287645 | -1.15302 |
| 12892540 | DMRT1 | doublesex and mab-3 related transcription factor 1 | 0.000103381 | 0.0173439 | 0.851382645 | -1.17456 |
| 12899957 | PERP | PERP, TP53 apoptosis effector | 0.000105136 | 0.0174711 | 0.788774166 | -1.26779 |
| 12908876 | MAGEE2 | melanoma antigen family E, 2 | 0.000105449 | 0.0174711 | 0.861898072 | -1.16023 |
| 12681492 | CCDC39 | coiled-coil domain containing 39 | 0.000108466 | 0.0178258 | 1.283912956 | 1.28391 |
| 12880093 | UNC13A | unc-13 homolog A (C. elegans) | 0.000108926 | 0.0178258 | 0.842268397 | -1.18727 |
| 12705423 | ANGPTL2 | angiopoietin-like 2 | 0.000111958 | 0.0182103 | 0.557637402 | -1.79328 |
| 12843233 | AMY2B | amylase, alpha 2B (pancreatic) | 0.000114346 | 0.0184009 | 1.275196285 | 1.2752 |
| 12735060 | SRM | spermidine synthase | 0.00011451 | 0.0184009 | 0.790713856 | -1.26468 |
| 12809293 | LAMA1 | laminin, alpha 1 | 0.000115227 | 0.0184053 | 1.402310166 | 1.40231 |
| 12738701 | SEC16B | SEC16 homolog B (S. cerevisiae) | 0.000117318 | 0.0186278 | 0.694391401 | -1.44011 |
| 12817661 | KDELR2 | KDEL (Lys-Asp-Glu-Leu) endoplasmic reticulum protein retentio | 0.000119433 | 0.0188194 | 0.880382438 | -1.13587 |
| 12891796 | PALM2 | paralemmin 2 | 0.000120481 | 0.0188194 | 1.200413903 | 1.20041 |
| 12782240 | CLASP1 | cytoplasmic linker associated protein 1 | 0.000121187 | 0.0188194 | 1.139714137 | 1.13971 |
| 12908168 | MAGED1 | melanoma antigen family D, 1 | 0.000121347 | 0.0188194 | 0.901233789 | -1.10959 |
| 12792677 | ITPK1 | inositol-tetrakisphosphate 1-kinase | 0.000122759 | 0.0189282 | 1.166775371 | 1.16677 |
| 12779964 | 4-Mar | membrane-associated ring finger (C3HC4) 4, E3 ubiquitin prote | 0.000125636 | 0.0191841 | 0.672133351 | -1.4878 |
| 12797483 | RBMS3 | RNA binding motif, single stranded interacting protein 3 | 0.000125856 | 0.0191841 | 1.173052176 | 1.17305 |
| 12895106 | ACER2 | alkaline ceramidase 2 | 0.000128917 | 0.0195389 | 0.770796078 | -1.29736 |
| 12889108 | CREB3 | cAMP responsive element binding protein 3 | 0.000130329 | 0.0196414 | 0.899029947 | -1.11231 |
| 12758956 | CMTM4 | CKLF-like MARVEL transmembrane domain containing 4 | 0.000135053 | 0.020239 | 1.161127315 | 1.16113 |
| 12692964 | C10H14orf119 | chromosome 10 open reading frame, human C14orf119 | 0.000136188 | 0.020295 | 0.865868336 | -1.15491 |
| 12786958 | ITGA2 | integrin, alpha 2 (CD49B, alpha 2 subunit of VLA-2 receptor) | 0.000137653 | 0.0203994 | 0.660161872 | -1.51478 |
| 12875311 | MAPK10 | mitogen-activated protein kinase 10 | 0.000139985 | 0.0204027 | 0.876255236 | -1.14122 |
| 12908904 | ZNF711 | zinc finger protein 711 | 0.000140058 | 0.0204027 | 1.102853191 | 1.10285 |
| 12767689 | ATP5G1 | ATP synthase, H+ transporting, mitochondrial Fo complex, subunit | 0.000140519 | 0.0204027 | 0.82350616 | -1.21432 |
| 12892113 | CNTNAP3 | contactin associated protein-like 3 | 0.000140735 | 0.0204027 | 1.622823388 | 1.62282 |
| 12849870 | TMEM213 | transmembrane protein 213 | 0.000141848 | 0.0204286 | 1.513237041 | 1.51324 |
| 12863307 | METTL21B | methyltransferase like 21B | 0.000142445 | 0.0204286 | 0.848716317 | -1.17825 |
| 12791435 | MIR380 | microRNA mir-380 | 0.000143776 | 0.0205093 | 0.584730352 | -1.71019 |
| 12678498 | APP | amyloid beta (A4) precursor protein | 0.000149384 | 0.0211959 | 1.122431456 | 1.12243 |
| 12865092 | RPS26 | ribosomal protein S26 | 0.000150367 | 0.0212104 | 0.728162409 | -1.37332 |
| 12689890 | MYO1E | myosin IE | 0.00015174 | 0.0212104 | 1.152660687 | 1.15266 |
| 12741977 | ADRBK2 | adrenergic, beta, receptor kinase 2 | 0.000152593 | 0.0212104 | 1.166377988 | 1.16638 |
| 12851480 | C4H7orf25 | chromosome 4 open reading frame, human C7orf25 | 0.000152767 | 0.0212104 | 0.800646923 | -1.24899 |
| 12710335 | LACC1 | laccase (multicopper oxidoreductase) domain containing 1 | 0.000153463 | 0.0212104 | 0.829689613 | -1.20527 |
| 12772085 | GPR142 | G protein-coupled receptor 142 | 0.000158547 | 0.0218002 | 0.815693952 | -1.22595 |
| 12710070 | COG3 | component of oligomeric golgi complex 3 | 0.000159708 | 0.0218472 | 0.919777046 | -1.08722 |
| 12697991 | RHOQ | ras homolog gene family, member Q | 0.000162447 | 0.0221085 | 0.790039186 | -1.26576 |
| 12822932 | SLC25A28 | solute carrier family 25, member 28 | 0.000164111 | 0.0222217 | 1.133959128 | 1.13396 |
| 12698264 | KCNIP3 | Kv channel interacting protein 3, calsenilin | 0.000167341 | 0.022473 | 0.548047307 | -1.82466 |
| 12900780 | SLC17A5 | solute carrier family 17 (anion | 0.000168625 | 0.022473 | 0.783293908 | -1.27666 |
| 12792547 | CCDC88C | coiled-coil domain containing 88C | 0.000168772 | 0.022473 | 1.191649398 | 1.19165 |
| 12679533 | TMEM45A | transmembrane protein 45A | 0.000169337 | 0.022473 | 0.721693381 | -1.38563 |
| 12691139 | PAK6 | p21 protein (Cdc42 | 0.000174499 | 0.02303 | 0.722982157 | -1.38316 |
| 12760883 | PRKCA | protein kinase C, alpha | 0.000175261 | 0.02303 | 0.768338315 | -1.30151 |
| 12821009 | KAZALD1 | Kazal-type serine peptidase inhibitor domain 1 | 0.000176297 | 0.0230525 | 0.664442997 | -1.50502 |
| 12782531 | PDK1 | pyruvate dehydrogenase kinase, isozyme 1 | 0.000180959 | 0.023272 | 0.927342699 | -1.07835 |
| 12892001 | KLHL9 | kelch-like 9 (Drosophila) | 0.000181304 | 0.023272 | 0.864296765 | -1.15701 |
| 12862551 | GXYLT1 | glucoside xylosyltransferase 1 | 0.000181488 | 0.023272 | 1.110395523 | 1.1104 |
| 12901117 | HEY2 | hairy | 0.000183368 | 0.023272 | 1.285266095 | 1.28527 |
| 12766850 | BT.62430 | angiotensin I converting enzyme | 0.000183914 | 0.023272 | 0.877878344 | -1.13911 |
| 12866811 | TAPBPL | TAP binding protein-like | 0.000184045 | 0.023272 | 1.184940824 | 1.18494 |
| 12755625 | HP | haptoglobin | 0.000184395 | 0.023272 | 0.471413486 | -2.12128 |
| 12847578 | LOC616625 | aquaporin 12B | 0.000186353 | 0.023272 | 0.745701034 | -1.34102 |
| 12890151 | SHC3 | SHC (Src homology 2 domain containing) transforming protein 3 | 0.000186712 | 0.023272 | 0.509126085 | -1.96415 |
| 12684272 | SLC25A36 | solute carrier family 25, member 36 | 0.000186931 | 0.023272 | 1.106474981 | 1.10647 |
| 12827916 | ZMYND17 | zinc finger, MYND-type containing 17 | 0.000187572 | 0.023272 | 1.118304293 | 1.1183 |
| 12915057 |  |  | 0.000193642 | 0.023814 | 1.184233588 | 1.18423 |
| 12841186 | SEC22B | SEC22 vesicle trafficking protein homolog B (S. cerevisiae) | 0.000194389 | 0.023814 | 0.904543522 | -1.10553 |
| 12749545 | BCAM | basal cell adhesion molecule (Lutheran blood group) | 0.000194619 | 0.023814 | 1.243713031 | 1.24371 |
| 12717993 | SVIL | supervillin | 0.000198067 | 0.0241252 | 0.704816008 | -1.41881 |
| 12910551 | ZBTB33 | zinc finger and BTB domain containing 33 | 0.000199082 | 0.0241387 | 0.883080184 | -1.1324 |
| 12874907 | CDKL2 | cyclin-dependent kinase-like 2 (CDC2-related kinase) | 0.000201696 | 0.0243449 | 0.804265826 | -1.24337 |
| 12687032 | PTTG1IP | pituitary tumor-transforming 1 interacting protein | 0.00020402 | 0.0243844 | 1.055259731 | 1.05526 |
| 12683071 | LSAMP | neuronal growth regulator 1-like | 0.000204208 | 0.0243844 | 1.356555759 | 1.35656 |
| 12716574 | GPR158 | G protein-coupled receptor 158 | 0.000204765 | 0.0243844 | 0.74012671 | -1.35112 |
| 12810467 | GALR1 | galanin receptor 1 | 0.000217049 | 0.0256058 | 0.630600521 | -1.58579 |
| 12809257 | COLEC12 | collectin sub-family member 12 | 0.000219757 | 0.0256058 | 1.294069538 | 1.29407 |
| 12749084 | LOC100336502 | aTPase, H+ | 0.000219966 | 0.0256058 | 1.365702299 | 1.3657 |
| 12724853 | EYA1 | eyes absent homolog 1 (Drosophila) | 0.000220297 | 0.0256058 | 1.265569671 | 1.26557 |
| 12801442 | SLC17A1 | solute carrier family 17 (sodium phosphate), member 1 | 0.000220419 | 0.0256058 | 0.595745188 | -1.67857 |
| 12794552 | GTDC2 | glycosyltransferase-like domain containing 2 | 0.000220781 | 0.0256058 | 0.909057852 | -1.10004 |
| 12865033 | BCL2L14 | BCL2-like 14 (apoptosis facilitator) | 0.000225534 | 0.0257878 | 0.738312513 | -1.35444 |
| 12773256 | LRRC46 | leucine rich repeat containing 46 | 0.000225769 | 0.0257878 | 0.782981122 | -1.27717 |
| 12771117 | PDK2 | pyruvate dehydrogenase kinase, isozyme 2 | 0.000226165 | 0.0257878 | 0.848968503 | -1.1779 |
| 12824808 | FGFR1 | fibroblast growth factor receptor 1 | 0.000226217 | 0.0257878 | 1.206971467 | 1.20697 |
| 12887654 | LYSMD3 | LysM, putative peptidoglycan-binding, domain containing 3 | 0.000230485 | 0.0261625 | 0.806659783 | -1.23968 |
| 12706345 | SEMA4C | sema domain, immunoglobulin domain (Ig), transmembrane domain | 0.000232746 | 0.0263072 | 1.191163946 | 1.19116 |
| 12703330 | PAIP2B | poly(A) binding protein interacting protein 2B | 0.000235805 | 0.0265405 | 1.222718102 | 1.22272 |
| 12853653 | YKT6 | YKT6 v-SNARE homolog (S. cerevisiae) | 0.000238511 | 0.0266211 | 0.846417538 | -1.18145 |
| 12885519 | SAR1B | SAR1 homolog B (S. cerevisiae) | 0.000238517 | 0.0266211 | 0.860985312 | -1.16146 |
| 12914859 |  |  | 0.000241756 | 0.0266288 | 0.864431247 | -1.15683 |
| 12782451 | 4-Mar | membrane-associated ring finger (C3HC4) 4, E3 ubiquitin prote | 0.000242022 | 0.0266288 | 0.734694477 | -1.36111 |
| 12742060 | SEC14L2 | SEC14-like 2 (S. cerevisiae) | 0.000242347 | 0.0266288 | 1.324896393 | 1.3249 |
| 12770437 | AMZ2 | archaelysin family metallopeptidase 2 | 0.000242579 | 0.0266288 | 0.894406382 | -1.11806 |
| 12769323 | CAMKK1 | calcium | 0.000243731 | 0.0266456 | 1.200349062 | 1.20035 |
| 12829453 | TM7SF2 | transmembrane 7 superfamily member 2 | 0.000246126 | 0.0267976 | 0.732681247 | -1.36485 |
| 12893064 | IL33 | interleukin 33 | 0.000249169 | 0.0270186 | 0.53793519 | -1.85896 |
| 12696484 | PNMA1 | paraneoplastic antigen MA1 | 0.000250658 | 0.0270701 | 0.829999502 | -1.20482 |
| 12682682 | LXN | latexin | 0.000252328 | 0.0270924 | 0.561665225 | -1.78042 |
| 12786382 | AMACR | alpha-methylacyl-CoA racemase | 0.000252896 | 0.0270924 | 0.772194809 | -1.29501 |
| 12882320 | GALNT10 | UDP-N-acetyl-alpha-D-galactosamine:polypeptide N-acetylgalac | 0.000254175 | 0.0271204 | 1.232255521 | 1.23226 |
| 12842993 | LMNA | lamin A | 0.000255266 | 0.0271245 | 0.843106341 | -1.18609 |
| 12771489 | SMTNL2 | smoothelin-like 2 | 0.000256501 | 0.0271245 | 1.243123971 | 1.24312 |
| 12816911 | GPRC5B | G protein-coupled receptor, family C, group 5, member B | 0.000257662 | 0.0271245 | 1.295618736 | 1.29562 |
| 12713584 | CHGB | chromogranin B (secretogranin 1) | 0.000261051 | 0.0271245 | 0.875013125 | -1.14284 |
| 12713592 | NPBWR2 | neuropeptides B | 0.000261347 | 0.0271245 | 0.822699937 | -1.21551 |
| 12827434 | USP54 | ubiquitin specific peptidase 54-like | 0.000261364 | 0.0271245 | 1.139607633 | 1.13961 |
| 12763243 | FLII | flightless I homolog (Drosophila) | 0.000263057 | 0.0271245 | 0.854525567 | -1.17024 |
| 12771261 | SGK494 | uncharacterized serine | 0.000263231 | 0.0271245 | 1.282812747 | 1.28281 |
| 12807700 | SEH1L | SEH1-like (S. cerevisiae) | 0.000263364 | 0.0271245 | 0.899871318 | -1.11127 |
| 12914903 |  |  | 0.000269858 | 0.0276864 | 0.922347559 | -1.08419 |
| 12857261 |  |  | 0.000272609 | 0.0278615 | 0.919793966 | -1.0872 |
| 12797969 | PLCD1 | phospholipase C, delta 1 | 0.000273898 | 0.0278806 | 1.210662547 | 1.21066 |
| 12850085 | SEMA3C | sema domain, immunoglobulin domain (Ig), short basic domain, | 0.000277219 | 0.0278806 | 1.411187613 | 1.41119 |
| 12773855 | SP140L | SP140 nuclear body protein-like | 0.000277568 | 0.0278806 | 1.197123074 | 1.19712 |
| 12914733 |  |  | 0.000277836 | 0.0278806 | 1.170401026 | 1.1704 |
| 12782828 | PLEKHM3 | pleckstrin homology domain containing, family M, member 3 | 0.000279063 | 0.0278806 | 1.208707529 | 1.20871 |
| 12786990 | PIK3R1 | phosphoinositide-3-kinase, regulatory subunit 1 (alpha) | 0.000279067 | 0.0278806 | 1.153558208 | 1.15356 |
| 12908260 | CA5B | carbonic anhydrase VB, mitochondrial | 0.000282571 | 0.027969 | 1.219067681 | 1.21907 |
| 12872493 | EMCN | endomucin | 0.000282722 | 0.027969 | 1.359140697 | 1.35914 |
| 12718182 | ATP5C1 | ATP synthase, H+ transporting, mitochondrial F1 complex, gamm | 0.000283098 | 0.027969 | 0.87744698 | -1.13967 |
| 12829389 | IGF2 | insulin-like growth factor 2 (somatomedin A) | 0.000286834 | 0.0279962 | 1.144147742 | 1.14415 |
| 12678652 | COPB2 | coatomer protein complex, subunit beta 2 (beta prime) | 0.000287375 | 0.0279962 | 0.907490426 | -1.10194 |
| 12734323 | DHRS3 | dehydrogenase | 0.000288108 | 0.0279962 | 1.212640075 | 1.21264 |
| 12791056 | GABRG3 | gamma-aminobutyric acid (GABA) A receptor, gamma 3 | 0.000288477 | 0.0279962 | 1.644139219 | 1.64414 |
| 12900549 | CITED2 | Cbp | 0.000288621 | 0.0279962 | 1.22554126 | 1.22554 |
| 12755952 | GOT2 | glutamic-oxaloacetic transaminase 2, mitochondrial (aspartate amin | 0.000291414 | 0.0281648 | 0.910158277 | -1.09871 |
| 12814236 | PPP1R35 | protein phosphatase 1, regulatory subunit 35 | 0.00029461 | 0.0283708 | 0.869860213 | -1.14961 |
| 12708563 |  |  | 0.00030118 | 0.0288992 | 0.782870787 | -1.27735 |
| 12754277 | FAM65A | family with sequence similarity 65, member A | 0.000306254 | 0.02923 | 0.807591359 | -1.23825 |
| 12833395 | CTSF | cathepsin F | 0.000306819 | 0.02923 | 1.121600569 | 1.1216 |
| 12730119 | MGC137098 | uncharacterized protein MGC137098 | 0.000313031 | 0.0297006 | 0.808106929 | -1.23746 |
| 12759391 | MEIS3 | Meis homeobox 3 | 0.000314998 | 0.0297006 | 0.752536046 | -1.32884 |
| 12732959 | KIF18A | kinesin family member 18A | 0.000315099 | 0.0297006 | 0.782013685 | -1.27875 |
| 12888569 | CDC42SE2 | CDC42 small effector 2 | 0.000317296 | 0.0298024 | 1.166652861 | 1.16665 |
| 12893911 | APBA1 | amyloid beta (A4) precursor protein-binding, family A, member | 0.000320335 | 0.0299822 | 1.184337375 | 1.18434 |
| 12726480 | MIR2318 | microRNA mir-2318 | 0.000323937 | 0.0301913 | 0.831234466 | -1.20303 |
| 12720297 | TRPC4AP | transient receptor potential cation channel, subfamily C, me | 0.00032673 | 0.0301913 | 0.892618049 | -1.1203 |
| 12800376 | PXK | PX domain containing serine | 0.00032878 | 0.0301913 | 0.834898769 | -1.19775 |
| 12893958 | GABBR2 | gamma-aminobutyric acid (GABA) B receptor, 2 | 0.000328872 | 0.0301913 | 1.3953404 | 1.39534 |
| 12897282 | REV3L | REV3-like, catalytic subunit of DNA polymerase zeta (yeast) | 0.000329791 | 0.0301913 | 1.147232359 | 1.14723 |
| 12789006 | CHGA | chromogranin A (parathyroid secretory protein 1) | 0.000330485 | 0.0301913 | 1.318871468 | 1.31887 |
| 12726663 | CD44 | CD44 molecule (Indian blood group) | 0.000331275 | 0.0301913 | 0.809742826 | -1.23496 |
| 12682117 | C1H21orf91 | chromosome 1 open reading frame, human C21orf91 | 0.000332146 | 0.0301913 | 0.734845646 | -1.36083 |
| 12678864 | HTR1F | 5-hydroxytryptamine (serotonin) receptor 1F | 0.000332755 | 0.0301913 | 0.352941592 | -2.83333 |
| 12723978 | MRPL33 | mitochondrial ribosomal protein L33 | 0.000334872 | 0.0302245 | 0.864012995 | -1.15739 |
| 12883172 | HAPLN1 | hyaluronan and proteoglycan link protein 1 | 0.00033692 | 0.0302245 | 0.732842329 | -1.36455 |
| 12888990 | BT.86327 | --- | 0.000337588 | 0.0302245 | 1.589499133 | 1.5895 |
| 12863567 | LOC100297468 | cD24 molecule-like | 0.000337821 | 0.0302245 | 0.579344067 | -1.72609 |
| 12793713 | SPTSSA | serine palmitoyltransferase, small subunit A | 0.000339183 | 0.0302245 | 0.893335716 | -1.1194 |
| 12725810 | CTHRC1 | collagen triple helix repeat containing 1 | 0.000339919 | 0.0302245 | 0.732960501 | -1.36433 |
| 12847281 | UQCRH | ubiquinol-cytochrome c reductase hinge protein | 0.000342227 | 0.0303286 | 0.896772516 | -1.11511 |
| 12828385 | OGDHL | oxoglutarate dehydrogenase-like | 0.000343961 | 0.030344 | 0.838033303 | -1.19327 |
| 12890349 | ZNF462 | zinc finger protein 462 | 0.000344676 | 0.030344 | 1.226116778 | 1.22612 |
| 12900759 | SLC18B1 | solute carrier family 18, subfamily B, member 1 | 0.000351444 | 0.030838 | 1.165346329 | 1.16535 |
| 12709313 | IPO5 | importin 5 | 0.000353896 | 0.0309374 | 0.884987079 | -1.12996 |
| 12834259 | PANX1 | pannexin 1 | 0.000355011 | 0.0309374 | 0.870450806 | -1.14883 |
| 12793325 | FBLN5 | fibulin 5 | 0.000356055 | 0.0309374 | 1.26675762 | 1.26676 |
| 12725994 | BAALC | brain and acute leukemia, cytoplasmic | 0.000358274 | 0.030978 | 0.833583408 | -1.19964 |
| 12735935 | SRGAP2 | SLIT-ROBO Rho GTPase activating protein 2 | 0.000359732 | 0.030978 | 1.136603873 | 1.1366 |
| 12867984 | TENC1 | tensin like C1 domain containing phosphatase (tensin 2) | 0.00036111 | 0.030978 | 1.189908151 | 1.18991 |
| 12752761 | BT.30326 | --- | 0.000361226 | 0.030978 | 0.883595172 | -1.13174 |
| 12720820 | C1QL3 | complement component 1, q subcomponent-like 3 | 0.000362329 | 0.030978 | 0.642153526 | -1.55726 |
| 12870236 | LPHN3 | latrophilin 3 | 0.000365286 | 0.031131 | 1.481084331 | 1.48109 |
| 12850899 | CTTNBP2 | cortactin binding protein 2 | 0.000369534 | 0.0311323 | 1.173783315 | 1.17378 |
| 12782838 | FAM126B | family with sequence similarity 126, member B | 0.000370213 | 0.0311323 | 0.877531679 | -1.13956 |
| 12720176 | FLRT3 | fibronectin leucine rich transmembrane protein 3 | 0.000370258 | 0.0311323 | 1.172379673 | 1.17238 |
| 12794461 | COPG1 | coatomer protein complex, subunit gamma | 0.00037049 | 0.0311323 | 0.887547706 | -1.1267 |
| 12843700 | SLC44A3 | solute carrier family 44, member 3 | 0.000371137 | 0.0311323 | 1.231869954 | 1.23187 |
| 12852942 | CFTR | cystic fibrosis transmembrane conductance regulator (ATP- | 0.000373023 | 0.0311924 | 0.702740689 | -1.423 |
| 12728764 | SCUBE2 | signal peptide, CUB domain and EGF like domain containing 2 | 0.000375739 | 0.0313214 | 1.255415549 | 1.25542 |
| 12861868 | RERG | RAS-like, estrogen-regulated, growth inhibitor | 0.000378144 | 0.031359 | 0.717252064 | -1.39421 |
| 12696911 | IGDCC4 | immunoglobulin superfamily, DCC subclass, member 4 | 0.000378541 | 0.031359 | 1.283947574 | 1.28395 |
| 12843378 | ATP6V0B | ATPase, H+ transporting, lysosomal 21kDa, V0 subunit b | 0.000381171 | 0.0314791 | 0.865239022 | -1.15575 |
| 12684999 | KCNJ6 | potassium inwardly-rectifying channel, subfamily J, member 6 | 0.000384105 | 0.0316235 | 0.671118419 | -1.49005 |
| 12874253 | GAR1 | GAR1 ribonucleoprotein homolog (yeast) | 0.000387854 | 0.0317025 | 0.776614582 | -1.28764 |
| 12827902 | MICU1 | mitochondrial calcium uptake 1 | 0.000387935 | 0.0317025 | 0.922773118 | -1.08369 |
| 12787557 | SH3PXD2B | SH3 and PX domains 2B | 0.000389192 | 0.0317025 | 1.181724396 | 1.18172 |
| 12762367 | MAP3K3 | mitogen-activated protein kinase kinase kinase 3 | 0.000389819 | 0.0317025 | 1.140518754 | 1.14052 |
| 12860901 | TMTC1 | transmembrane and tetratricopeptide repeat containing 1 | 0.0003924 | 0.0318053 | 1.287070858 | 1.28707 |
| 12706012 | EFR3B | EFR3 homolog B (S. cerevisiae) | 0.000394475 | 0.0318053 | 0.716794495 | -1.3951 |
| 12886767 | KXD1 | KxDL motif containing 1 | 0.000395284 | 0.0318053 | 0.882822915 | -1.13273 |
| 12803164 | GNMT | glycine N-methyltransferase | 0.000395853 | 0.0318053 | 0.832015975 | -1.2019 |
| 12693805 | RPS6KA5 | ribosomal protein S6 kinase, 90kDa, polypeptide 5 | 0.000399962 | 0.032039 | 1.180848061 | 1.18085 |
| 12756690 |  |  | 0.000405224 | 0.0323633 | 0.789540172 | -1.26656 |
| 12681327 | DYRK1A | dual-specificity tyrosine-(Y)-phosphorylation regulated kinas | 0.000407106 | 0.0324166 | 1.099331167 | 1.09933 |
| 12748294 | ZNF605 | zinc finger protein 605 | 0.000409743 | 0.0325294 | 1.119083963 | 1.11908 |
| 12861444 | SYT10 | synaptotagmin X | 0.000412261 | 0.0326171 | 1.384230569 | 1.38423 |
| 12867796 | ACVR1B | activin A receptor, type IB | 0.000413292 | 0.0326171 | 1.118209255 | 1.11821 |
| 12911995 |  |  | 0.000414571 | 0.0326215 | 1.171847993 | 1.17185 |
| 12838286 | NOTCH2 | notch 2 | 0.000416917 | 0.0327096 | 1.201684762 | 1.20169 |
| 12711493 | FRY | furry homolog (Drosophila) | 0.000419974 | 0.0328111 | 1.190640612 | 1.19064 |
| 12885485 | GLRX | glutaredoxin (thioltransferase) | 0.000421727 | 0.0328111 | 0.836120401 | -1.196 |
| 12782135 | ERBB4 | erb-b2 receptor tyrosine kinase 4 | 0.000421901 | 0.0328111 | 1.254325856 | 1.25433 |
| 12868475 | ANKS1B | ankyrin repeat and sterile alpha motif domain containing 1B | 0.000424675 | 0.0329308 | 1.225704995 | 1.2257 |
| 12873141 | BTC | betacellulin | 0.000427725 | 0.0330202 | 1.418270731 | 1.41827 |
| 12885399 | ERAP1 | endoplasmic reticulum aminopeptidase 1 | 0.000428303 | 0.0330202 | 1.25194521 | 1.25195 |
| 12896756 | QKI | QKI, KH domain containing, RNA binding | 0.000429992 | 0.0330549 | 1.141169516 | 1.14117 |
| 12774157 | CNTNAP5 | contactin associated protein-like 5 | 0.000434841 | 0.0333316 | 1.387898084 | 1.3879 |
| 12899842 | MOXD1 | monooxygenase, DBH-like 1 | 0.00043675 | 0.0333433 | 0.625480838 | -1.59877 |
| 12858199 | LRIG3 | leucine-rich repeats and immunoglobulin-like domains 3 | 0.000438412 | 0.0333433 | 1.294873853 | 1.29487 |
| 12866171 | GOLT1B | golgi transport 1B | 0.000438744 | 0.0333433 | 0.86948205 | -1.15011 |
| 12812980 | RASA4 | RAS p21 protein activator 4 | 0.000440354 | 0.0333706 | 1.145554105 | 1.14555 |
| 12790698 | PPP4R4 | protein phosphatase 4, regulatory subunit 4 | 0.000441766 | 0.0333827 | 1.247242036 | 1.24724 |
| 12778629 | WIPF1 | WAS | 0.000444035 | 0.0334594 | 1.192497758 | 1.1925 |
| 12706299 | NCOA1 | nuclear receptor coactivator 1 | 0.000446813 | 0.0335739 | 1.12335064 | 1.12335 |
| 12867807 | CSRP2 | cysteine and glycine-rich protein 2 | 0.000448135 | 0.0335786 | 1.17811718 | 1.17812 |
| 12790124 | ALPK3 | alpha-kinase 3 | 0.000450127 | 0.0336334 | 0.79828847 | -1.25268 |
| 12815419 | COG7 | component of oligomeric golgi complex 7 | 0.000455987 | 0.0339761 | 0.866310902 | -1.15432 |
| 12900189 | DSE | dermatan sulfate epimerase | 0.000461485 | 0.03429 | 0.774077686 | -1.29186 |
| 12911809 |  |  | 0.000467454 | 0.034637 | 1.240011706 | 1.24001 |
| 12826094 | SEC24C | SEC24 family, member C (S. cerevisiae) | 0.000469176 | 0.0346683 | 0.891503967 | -1.1217 |
| 12869248 | ERBB3 | v-erb-b2 erythroblastic leukemia viral oncogene homolog 3 (avi | 0.000472712 | 0.0348331 | 1.190114906 | 1.19011 |
| 12834682 | HTATIP2 | HIV-1 Tat interactive protein 2, 30kDa | 0.00047662 | 0.0350243 | 0.861690119 | -1.16051 |
| 12859857 | PLXNC1 | plexin C1 | 0.000481822 | 0.0353093 | 1.259598138 | 1.2596 |
| 12744300 | FBXW7 | F-box and WD repeat domain containing 7 | 0.000484596 | 0.0354153 | 0.870708496 | -1.14849 |
| 12889697 | RUSC2 | RUN and SH3 domain containing 2 | 0.000486642 | 0.0354677 | 0.838391629 | -1.19276 |
| 12865601 | MARS | methionyl-tRNA synthetase | 0.000490565 | 0.0354978 | 0.866626224 | -1.1539 |
| 12850206 | AGK | acylglycerol kinase | 0.000490801 | 0.0354978 | 0.877454679 | -1.13966 |
| 12907940 | PDK3 | pyruvate dehydrogenase kinase, isozyme 3 | 0.000492327 | 0.0354978 | 0.863490748 | -1.15809 |
| 12881048 | C7H5orf30 | UNC119-binding protein C5orf30 homolog | 0.000492818 | 0.0354978 | 0.853890753 | -1.17111 |
| 12875407 | RRH | retinal pigment epithelium-derived rhodopsin homolog | 0.000494206 | 0.0354978 | 1.679247026 | 1.67925 |
| 12804713 | RPS4Y1 | ribosomal protein S4, Y-linked 1 | 0.00049504 | 0.0354978 | 0.83037857 | -1.20427 |
| 12786020 | C1QTNF3 | C1q and tumor necrosis factor related protein 3 | 0.000497789 | 0.0355992 | 0.548380905 | -1.82355 |
| 12772050 | KIF19 | kinesin family member 19 | 0.000501395 | 0.0357612 | 1.367467136 | 1.36747 |
| 12862410 | PARVB | parvin, beta | 0.000503447 | 0.035779 | 0.525041872 | -1.90461 |
| 12712868 | MRP63 | mitochondrial ribosomal protein 63 | 0.000504327 | 0.035779 | 0.850918993 | -1.1752 |
| 12850925 | NACAD | NAC alpha domain containing | 0.000508742 | 0.0358703 | 0.843198759 | -1.18596 |
| 12842417 | LOC100298793 | cytochrome P450, family 2, subfamily J, polypeptide 2-l | 0.000511358 | 0.0358703 | 1.419617697 | 1.41962 |
| 12851927 | NME2 | non-metastatic cells 2, protein (NM23B) expressed in | 0.000511441 | 0.0358703 | 0.842829209 | -1.18648 |
| 12711284 | GAS6 | growth arrest-specific 6 | 0.000512288 | 0.0358703 | 1.225511222 | 1.22551 |
| 12894461 | CHRNA2 | cholinergic receptor, nicotinic, alpha 2 (neuronal) | 0.000512337 | 0.0358703 | 0.635849177 | -1.5727 |
| 12914639 |  |  | 0.000514411 | 0.0359212 | 1.155110556 | 1.15511 |
| 12892374 | LOC100295263 | uncharacterized LOC100295263 | 0.000516545 | 0.0359281 | 1.11403111 | 1.11403 |
| 12746149 | SLC24A6 | solute carrier family 24 (sodium | 0.000517204 | 0.0359281 | 1.179477562 | 1.17948 |
| 12860410 | EFCAB4B | EF-hand calcium binding domain 4B | 0.000523419 | 0.0362076 | 1.277811569 | 1.27781 |
| 12740926 | DUSP10 | dual specificity phosphatase 10 | 0.000527983 | 0.0362076 | 1.221833679 | 1.22183 |
| 12857795 | AGAP2 | ArfGAP with GTPase domain, ankyrin repeat and PH domain 2 | 0.000528276 | 0.0362076 | 1.240577812 | 1.24058 |
| 12771875 | SRCIN1 | SRC kinase signaling inhibitor 1 | 0.000528292 | 0.0362076 | 1.208958869 | 1.20896 |
| 12898982 | MGC127538 | uncharacterized protein MGC127538 | 0.000529202 | 0.0362076 | 0.669841716 | -1.49289 |
| 12710496 | DCLK1 | doublecortin-like kinase 1 | 0.000529371 | 0.0362076 | 0.753698777 | -1.32679 |
| 12765386 | SLC39A11 | solute carrier family 39 (metal ion transporter), member 11 | 0.000531096 | 0.0362327 | 0.822882723 | -1.21524 |
| 12723165 | ADCY8 | adenylate cyclase 8 (brain) | 0.000534084 | 0.0363436 | 0.791114205 | -1.26404 |
| 12901045 | MAP3K5 | mitogen-activated protein kinase kinase kinase 5 | 0.000537613 | 0.0364217 | 1.298559638 | 1.29856 |
| 12740562 | EPRS | glutamyl-prolyl-tRNA synthetase | 0.000537963 | 0.0364217 | 0.890749566 | -1.12265 |
| 12822071 | MYOF | myoferlin | 0.000542297 | 0.0366068 | 1.271674095 | 1.27167 |
| 12698500 | CNGA3 | cyclic nucleotide gated channel alpha 3 | 0.000543441 | 0.0366068 | 0.750632408 | -1.33221 |
| 12710994 | SERTM1 | serine-rich and transmembrane domain containing 1 | 0.000548783 | 0.0367467 | 1.646334436 | 1.64633 |
| 12822230 | ALDH18A1 | aldehyde dehydrogenase 18 family, member A1 | 0.000549551 | 0.0367467 | 0.890376807 | -1.12312 |
| 12728517 | LDLRAD3 | low density lipoprotein receptor class A domain containing 3 | 0.000552262 | 0.0367467 | 1.193025573 | 1.19303 |
| 12850342 | TNS3 | tensin 3 | 0.000552327 | 0.0367467 | 1.132020793 | 1.13202 |
| 12767297 | ARRB2 | arrestin, beta 2 | 0.000552406 | 0.0367467 | 1.205350793 | 1.20535 |
| 12692077 | RAD51B | RAD51 homolog B (S. cerevisiae) | 0.000557082 | 0.0368933 | 1.175414098 | 1.17541 |
| 12811667 | ACTL6B | actin-like 6B | 0.000557477 | 0.0368933 | 0.88520643 | -1.12968 |
| 12784195 | WDFY1 | WD repeat and FYVE domain containing 1 | 0.000558759 | 0.0368933 | 1.167194822 | 1.16719 |
| 12856701 | CS | citrate synthase | 0.000562572 | 0.0370533 | 0.908694388 | -1.10048 |
| 12695573 | LOC527711 | spectrin beta chain, erythrocyte-like | 0.000569403 | 0.0374109 | 0.835470746 | -1.19693 |
| 12721237 | JAG1 | jagged 1 | 0.000571214 | 0.0374377 | 1.236745183 | 1.23675 |
| 12699180 | CRIM1 | cysteine rich transmembrane BMP regulator 1 (chordin-like) | 0.000575076 | 0.0375984 | 1.227322155 | 1.22732 |
| 12755154 |  |  | 0.000578463 | 0.037616 | 0.816526496 | -1.2247 |
| 12883643 | SLCO4C1 | solute carrier organic anion transporter family, member 4C1 | 0.000579234 | 0.037616 | 1.512333076 | 1.51233 |
| 12786303 |  |  | 0.000580875 | 0.037616 | 0.86613082 | -1.15456 |
| 12857841 | AMDHD1 | amidohydrolase domain containing 1 | 0.000580986 | 0.037616 | 1.271539886 | 1.27154 |
| 12692448 | B2M | beta-2-microglobulin | 0.000589776 | 0.0380545 | 1.232003509 | 1.232 |
| 12753406 | SYT5 | synaptotagmin V | 0.000590611 | 0.0380545 | 0.881694264 | -1.13418 |
| 12896514 | ESR1 | estrogen receptor 1 | 0.000595193 | 0.0382573 | 1.23512447 | 1.23512 |
| 12759210 | U2AF1L4 | U2 small nuclear RNA auxiliary factor 1-like 4 | 0.000598874 | 0.0383285 | 1.111858527 | 1.11186 |
| 12879784 | PRELID1 | PRELI domain containing 1 | 0.000599175 | 0.0383285 | 0.903481113 | -1.10683 |
| 12788870 | MIR329B | microRNA mir-329b | 0.000604208 | 0.0384602 | 0.644932443 | -1.55055 |
| 12773652 | LEPREL4 | leprecan-like 4 | 0.00060462 | 0.0384602 | 0.87152805 | -1.14741 |
| 12899728 | PGM3 | phosphoglucomutase 3 | 0.000605559 | 0.0384602 | 0.862887221 | -1.1589 |
| 12875625 | SMIM14 | small integral membrane protein 14 | 0.000612085 | 0.0386216 | 0.895070845 | -1.11723 |
| 12846438 | UAP1 | UDP-N-acteylglucosamine pyrophosphorylase 1 | 0.000613386 | 0.0386216 | 0.840830741 | -1.1893 |
| 12880280 | BT.34956 | --- | 0.00061365 | 0.0386216 | 0.81617329 | -1.22523 |
| 12854454 | RAPGEF5 | Rap guanine nucleotide exchange factor (GEF) 5 | 0.000613892 | 0.0386216 | 1.30135471 | 1.30136 |
| 12725367 | GRHL2 | grainyhead-like 2 (Drosophila) | 0.000621379 | 0.0388747 | 1.251741485 | 1.25174 |
| 12880370 | PCDHGB4 | protocadherin gamma subfamily B, 4 | 0.000621625 | 0.0388747 | 1.152806854 | 1.15281 |
| 12700679 | NBAS | neuroblastoma amplified sequence | 0.000622945 | 0.0388747 | 0.882005327 | -1.13378 |
| 12675621 |  |  | 0.000624917 | 0.0388747 | 1.576764794 | 1.57677 |
| 12696845 | SLC8A3 | solute carrier family 8 (sodium | 0.000625201 | 0.0388747 | 0.655849522 | -1.52474 |
| 12807569 | ST8SIA3 | ST8 alpha-N-acetyl-neuraminide alpha-2,8-sialyltransfe | 0.000629731 | 0.039007 | 1.140526558 | 1.14053 |
| 12864912 | SHMT2 | serine hydroxymethyltransferase 2 (mitochondrial) | 0.000630253 | 0.039007 | 0.837527952 | -1.19399 |
| 12752645 | SLC7A6 | solute carrier family 7 (amino acid transporter light chain, | 0.00063368 | 0.0391193 | 0.845437176 | -1.18282 |
| 12686282 | FAM43A | family with sequence similarity 43, member A | 0.000635002 | 0.0391193 | 1.310209018 | 1.31021 |
| 12779984 | MIR2356 | microRNA mir-2356 | 0.000641561 | 0.0394323 | 1.375402305 | 1.3754 |
| 12831806 | SYTL2 | synaptotagmin-like 2 | 0.000644972 | 0.0395509 | 0.616614049 | -1.62176 |
| 12857257 | SLC25A3 | solute carrier family 25 (mitochondrial carrier; phosphate carr | 0.000648197 | 0.0395957 | 0.912508669 | -1.09588 |
| 12714175 | PRKCQ | protein kinase C, theta | 0.000649741 | 0.0395957 | 0.680804711 | -1.46885 |
| 12834738 | ZBTB44 | zinc finger and BTB domain containing 44 | 0.000650209 | 0.0395957 | 1.095670676 | 1.09567 |
| 12898481 | EYA4 | eyes absent homolog 4 (Drosophila) | 0.00065164 | 0.0395957 | 1.30495832 | 1.30496 |
| 12824728 | MIR2400 | microRNA mir-2400 | 0.00065673 | 0.0397483 | 1.079059449 | 1.07906 |
| 12683496 | ITGB5 | integrin, beta 5 | 0.000657132 | 0.0397483 | 1.246624763 | 1.24662 |
| 12709172 | MCF2L | MCF.2 cell line derived transforming sequence-like | 0.000659204 | 0.0397834 | 1.147817024 | 1.14782 |
| 12789506 | PDE8A | phosphodiesterase 8A | 0.00066136 | 0.0398234 | 0.855073579 | -1.16949 |
| 12813668 | CALN1 | calneuron 1 | 0.000663369 | 0.0398544 | 1.292863265 | 1.29286 |
| 12874507 | NSG1 | neuron specific gene family member 1 | 0.000667118 | 0.0399896 | 1.246686929 | 1.24669 |
| 12811457 | DNASE1 | deoxyribonuclease I | 0.000669711 | 0.040055 | 1.104228089 | 1.10423 |
| 12765442 | YPEL2 | yippee-like 2 (Drosophila) | 0.000675232 | 0.0402949 | 0.797384579 | -1.2541 |
| 12705319 | CHST10 | carbohydrate sulfotransferase 10 | 0.000676835 | 0.0403004 | 0.872965989 | -1.14552 |
| 12906927 | TBC1D8B | TBC1 domain family, member 8B (with GRAM domain) | 0.000679038 | 0.0403415 | 0.870958751 | -1.14816 |
| 12803392 | SLC39A7 | solute carrier family 39 (zinc transporter), member 7 | 0.000683522 | 0.0405177 | 0.878757788 | -1.13797 |
| 12851829 | AMPH | amphiphysin | 0.000687146 | 0.0405511 | 1.255626777 | 1.25563 |
| 12841921 | LOC100848786 | lipid phosphate phosphatase-related protein type 5-like | 0.000687415 | 0.0405511 | 1.290663982 | 1.29066 |
| 12890580 | CNTRL | centriolin | 0.000688646 | 0.0405511 | 1.162593327 | 1.16259 |
| 12859128 | C1R | complement component 1, r subcomponent | 0.000690455 | 0.040568 | 1.266630863 | 1.26663 |
| 12863934 | BTG1 | B-cell translocation gene 1, anti-proliferative | 0.000695771 | 0.0407905 | 1.21894286 | 1.21894 |
| 12879860 | RASGRF2 | Ras protein-specific guanine nucleotide-releasing factor 2 | 0.00070411 | 0.0411889 | 1.15565787 | 1.15566 |
| 12698459 | POMC | proopiomelanocortin | 0.000711262 | 0.0415162 | 0.797441807 | -1.25401 |
| 12731200 | LGR4 | leucine-rich repeat containing G protein-coupled receptor 4 | 0.000714431 | 0.0415858 | 1.135924756 | 1.13593 |
| 12711615 | COG6 | component of oligomeric golgi complex 6 | 0.000716027 | 0.0415858 | 0.909173561 | -1.0999 |
| 12845308 | TCTEX1D1 | Tctex1 domain containing 1 | 0.000717131 | 0.0415858 | 1.402495039 | 1.4025 |
| 12884927 | PJA2 | praja ring finger 2 | 0.000721913 | 0.0416887 | 0.919438775 | -1.08762 |
| 12750642 | TERF2IP | telomeric repeat binding factor 2, interacting protein | 0.000722031 | 0.0416887 | 0.845408586 | -1.18286 |
| 12803365 |  |  | 0.000730378 | 0.0420796 | 1.115262365 | 1.11526 |
| 12676495 |  |  | 0.000734621 | 0.0422328 | 1.381036164 | 1.38104 |
| 12691075 | SV2C | synaptic vesicle glycoprotein 2C | 0.000740752 | 0.0424937 | 0.453570278 | -2.20473 |
| 12900080 | TCP1 | t-complex 1 | 0.000745803 | 0.0426916 | 0.857728563 | -1.16587 |
| 12780180 | PKP4 | plakophilin 4 | 0.000757244 | 0.0431826 | 1.084579877 | 1.08458 |
| 12715823 | ARMC4 | armadillo repeat containing 4 | 0.000757617 | 0.0431826 | 1.168029173 | 1.16803 |
| 12861001 | SOX5 | SRY (sex determining region Y)-box 5 | 0.000759798 | 0.0432145 | 1.193276128 | 1.19328 |
| 12746075 | RFC5 | replication factor C (activator 1) 5, 36.5kDa | 0.000762194 | 0.0432236 | 1.077600141 | 1.0776 |
| 12801783 | LOC512672 | major histocompatibility complex, class I | 0.000763198 | 0.0432236 | 1.198532038 | 1.19853 |
| 12812837 | ERCC4 | excision repair cross-complementing rodent repair deficiency, | 0.00076984 | 0.0435074 | 1.096765419 | 1.09677 |
| 12695501 | SCG3 | secretogranin III | 0.000774345 | 0.0436695 | 0.776934372 | -1.28711 |
| 12752141 | MTSS1L | metastasis suppressor 1-like | 0.000777449 | 0.0437029 | 0.815281639 | -1.22657 |
| 12826866 | LRRTM3 | leucine rich repeat transmembrane neuronal 3 | 0.000779829 | 0.0437029 | 1.57385388 | 1.57385 |
| 12782645 | PADI6 | peptidyl arginine deiminase, type VI | 0.000779854 | 0.0437029 | 0.741383273 | -1.34883 |
| 12688919 | LYSMD2 | LysM, putative peptidoglycan-binding, domain containing 2 | 0.000785376 | 0.0437927 | 0.785040273 | -1.27382 |
| 12904088 | ALG13 | asparagine-linked glycosylation 13 homolog (S. cerevisiae) | 0.000787521 | 0.0437927 | 1.221019111 | 1.22102 |
| 12840703 | C3H1orf111 | chromosome 3 open reading frame, human C1orf111 | 0.000787783 | 0.0437927 | 0.76661837 | -1.30443 |
| 12792023 | FES | feline sarcoma oncogene | 0.000790434 | 0.0437927 | 1.212207903 | 1.21221 |
| 12722327 | TSTA3 | tissue specific transplantation antigen P35B | 0.000794299 | 0.0437927 | 0.843355204 | -1.18574 |
| 12835758 | SV2A | synaptic vesicle glycoprotein 2A | 0.00079551 | 0.0437927 | 1.194972988 | 1.19497 |
| 12723553 | DEPTOR | DEP domain containing MTOR-interacting protein | 0.000795644 | 0.0437927 | 1.159042677 | 1.15904 |
| 12914751 |  |  | 0.000796285 | 0.0437927 | 0.751738395 | -1.33025 |
| 12908846 | GABRE | gamma-aminobutyric acid (GABA) A receptor, epsilon | 0.000798033 | 0.0437927 | 1.281408935 | 1.28141 |
| 12716263 | RPN2 | ribophorin II | 0.00079843 | 0.0437927 | 0.885927921 | -1.12876 |
| 12911099 |  |  | 0.000802964 | 0.0437927 | 1.160710866 | 1.16071 |
| 12876698 | C3 | complement component 3 | 0.000803048 | 0.0437927 | 1.471232982 | 1.47123 |
| 12740723 | LOC539953 | denticleless protein homolog | 0.00080336 | 0.0437927 | 0.738029167 | -1.35496 |
| 12849407 | MEST | mesoderm specific transcript homolog (mouse) | 0.00080444 | 0.0437927 | 0.843540169 | -1.18548 |
| 12715842 | TTPAL | tocopherol (alpha) transfer protein-like | 0.000810652 | 0.0439839 | 0.888754588 | -1.12517 |
| 12825112 | WHSC1L1 | Wolf-Hirschhorn syndrome candidate 1-like 1 | 0.000811249 | 0.0439839 | 1.069198529 | 1.0692 |
| 12828947 | JMJD1C | jumonji domain containing 1C | 0.000817653 | 0.0442412 | 1.10987545 | 1.10988 |
| 12678271 | TMEM207 | transmembrane protein 207 | 0.000834653 | 0.0448318 | 0.863490748 | -1.15809 |
| 12902219 | GPRASP1 | G protein-coupled receptor associated sorting protein 1 | 0.000835727 | 0.0448318 | 0.837773533 | -1.19364 |
| 12824828 | SFRP1 | secreted frizzled-related protein 1 | 0.000839379 | 0.0448318 | 1.233955494 | 1.23395 |
| 12818938 | GRK5 | G protein-coupled receptor kinase 5 | 0.000840328 | 0.0448318 | 1.185206256 | 1.18521 |
| 12873401 | BMPR1B | bone morphogenetic protein receptor, type IB | 0.000841157 | 0.0448318 | 1.246388589 | 1.24639 |
| 12873854 | PLAC8 | placenta-specific 8 | 0.000841599 | 0.0448318 | 1.776075902 | 1.77608 |
| 12808035 | CHST9 | carbohydrate (N-acetylgalactosamine 4-0) sulfotransferase 9 | 0.000841858 | 0.0448318 | 0.825157399 | -1.21189 |
| 12790928 | LRFN5 | leucine rich repeat and fibronectin type III domain containing | 0.000842015 | 0.0448318 | 1.44951905 | 1.44952 |
| 12875951 | C6H4orf32 | chromosome 6 open reading frame, human C4orf32 | 0.000845028 | 0.0449026 | 0.715358752 | -1.3979 |
| 12861802 | PARP11 | poly (ADP-ribose) polymerase family, member 11 | 0.000847815 | 0.0449612 | 1.109924725 | 1.10992 |
| 12897014 | FRK | fyn-related kinase | 0.000849767 | 0.0449753 | 1.359885552 | 1.35989 |
| 12896683 | BCKDHB | branched chain keto acid dehydrogenase E1, beta polypep | 0.00085525 | 0.0449922 | 0.87024628 | -1.1491 |
| 12786321 | ESM1 | endothelial cell-specific molecule 1 | 0.000856092 | 0.0449922 | 0.55619518 | -1.79793 |
| 12824753 | PLAT | plasminogen activator, tissue | 0.000856745 | 0.0449922 | 1.26614974 | 1.26615 |
| 12825144 | PPP1R3B | protein phosphatase 1, regulatory subunit 3B | 0.000856834 | 0.0449922 | 0.775716956 | -1.28913 |
| 12698660 | EPAS1 | endothelial PAS domain protein 1 | 0.000859307 | 0.0450334 | 1.238233684 | 1.23823 |
| 12842746 | ATP1A1 | ATPase, Na+ | 0.000871202 | 0.0455673 | 0.890971783 | -1.12237 |
| 12692159 | REEP5 | receptor accessory protein 5 | 0.000873341 | 0.0455898 | 0.932392239 | -1.07251 |
| 12787195 | SLC30A5 | solute carrier family 30 (zinc transporter), member 5 | 0.000876799 | 0.0456334 | 0.911294585 | -1.09734 |
| 12915041 |  |  | 0.000877599 | 0.0456334 | 0.763918597 | -1.30904 |
| 12749816 | GAS8 | growth arrest-specific 8 | 0.000892365 | 0.0462828 | 1.171628317 | 1.17163 |
| 12892286 | STOM | stomatin | 0.000894615 | 0.0462828 | 1.156473127 | 1.15647 |
| 12832646 | SRPR | signal recognition particle receptor (docking protein) | 0.000895292 | 0.0462828 | 0.862522533 | -1.15939 |
| 12875956 | PLAC8 | placenta-specific 8 | 0.000899949 | 0.0464335 | 1.357144893 | 1.35714 |
| 12829383 | GNG3 | guanine nucleotide binding protein (G protein), gamma 3 | 0.000903666 | 0.0465251 | 0.726738905 | -1.37601 |
| 12741898 | CPE | carboxypeptidase E | 0.000905212 | 0.0465251 | 0.905764284 | -1.10404 |
| 12897969 | BAI3 | brain-specific angiogenesis inhibitor 3 | 0.00090769 | 0.0465627 | 1.21778502 | 1.21778 |
| 12915157 |  |  | 0.000914302 | 0.046758 | 0.82361468 | -1.21416 |
| 12807710 | FAM59A | family with sequence similarity 59, member A | 0.000915002 | 0.046758 | 1.113502667 | 1.1135 |
| 12915155 |  |  | 0.000919174 | 0.0468174 | 0.845129939 | -1.18325 |
| 12788800 | MIR544A | microRNA mir-544a | 0.000919675 | 0.0468174 | 0.616431601 | -1.62224 |
| 12747065 | MSI1 | musashi homolog 1 (Drosophila) | 0.000926472 | 0.0469521 | 1.245961527 | 1.24596 |
| 12910177 | MID1IP1 | MID1 interacting protein 1 (gastrulation specific G12 homolo | 0.000931189 | 0.0469521 | 1.144915374 | 1.14492 |
| 12830490 | LOC512612 | histone H2B type 1-like | 0.00093122 | 0.0469521 | 1.153970871 | 1.15397 |
| 12794684 | CSPG5 | chondroitin sulfate proteoglycan 5 (neuroglycan C) | 0.000931634 | 0.0469521 | 1.378454752 | 1.37846 |
| 12787836 | PELO | pelota homolog (Drosophila) | 0.000932739 | 0.0469521 | 0.887839266 | -1.12633 |
| 12682282 | APOD | apolipoprotein D | 0.000934393 | 0.0469521 | 1.471865295 | 1.47186 |
| 12836105 | GNG12 | guanine nucleotide binding protein (G protein), gamma 12 | 0.000934642 | 0.0469521 | 0.817795224 | -1.2228 |
| 12834462 | PITPNM1 | phosphatidylinositol transfer protein, membrane-associated 1 | 0.000938923 | 0.0470785 | 0.827876249 | -1.20791 |
| 12914259 |  |  | 0.000944074 | 0.0471625 | 0.793197538 | -1.26072 |
| 12814004 | TMEM204 | transmembrane protein 204 | 0.000944133 | 0.0471625 | 1.160667755 | 1.16067 |
| 12679678 | PLCL2 | phospholipase C-like 2 | 0.000948677 | 0.0473009 | 1.155484275 | 1.15548 |
| 12765359 | FAM104A | family with sequence similarity 104, member A | 0.000952402 | 0.0473137 | 0.900495272 | -1.1105 |
| 12691043 | TTLL5 | tubulin tyrosine ligase-like family, member 5 | 0.000952481 | 0.0473137 | 1.13304048 | 1.13304 |
| 12839347 | WLS | wntless homolog (Drosophila) | 0.000960055 | 0.0476013 | 1.161903058 | 1.1619 |
| 12761717 | MSI2 | musashi homolog 2 (Drosophila) | 0.000968534 | 0.0479326 | 0.942871421 | -1.06059 |
| 12729022 | MADD | MAP-kinase activating death domain | 0.000972831 | 0.0480561 | 0.872638422 | -1.14595 |
| 12875914 | ABLIM2 | actin binding LIM protein family member 2 | 0.0009844 | 0.0485377 | 1.292654362 | 1.29265 |
| 12700884 | CCDC85A | coiled-coil domain containing 85A | 0.000995188 | 0.048979 | 1.252536386 | 1.25254 |
