# Supplementary material for: Pituitary genomic expression profiles of steers are altered by grazing of high vs. low endophyte-infected tall fescue forages
Source: PLoS One. 2017 Sep 13;12(9):e0184612. doi: 10.1371/journal.pone.0184612 (PMC5597216; doi:10.1371/journal.pone.0184612)
Supplement: S3 Table — (DOCX) [file pone.0184612.s006.docx]

**Supplemental Table 3.** List of selected genes involved in prolactin or POMC/ACTH expression expressed by pituitaries collected from steers grazing high- (HE, n = 8) or low- (LE, n = 8) endophyte-infected forages.

| Transcript ID | Gene Symbol | Gene_assignment | p-value | False Discovery Rate | Ratio(HE vs.LE) | Fold-Change(HE vs. LE) |
| --- | --- | --- | --- | --- | --- | --- |
| 12730735 | DRD2 | Dopamine receptor D2 | 5.55E-07 | 0.00148011 | 0.569216758 | -1.7568 |
| 12804415 | PRL | Prolactin | 6.12E-06 | 0.00411905 | 0.814617496 | -1.22757 |
| 12785083 | PRLR | Prolactin receptor | 8.86E-05 | 0.0163351 | 0.762951095 | -1.3107 |
| 12683485 | POU1F1 | POU class 1 homeobox 1 | 0.00350045 | 0.0919041 | 0.769538585 | -1.29948 |
| 12829370 | GAL | Galanin/GMAP prepropeptide | 0.00857877 | 0.143484 | 0.745089858 | -1.34212 |
| 12896608 | VIP | Vasoactive intestinal peptide | 0.00330761 | 0.0891485 | 0.568424044 | -1.75925 |
| 12698459 | POMC | Proopiomelanocortin | 0.000711262 | 0.0415162 | 0.797441807 | -1.25401 |
| 12883237 | PCSK1 | Proprotein convertase subtilisin/kexin type 1 | 3.55E-06 | 0.00326731 | 0.580548154 | -1.72251 |
| 12766978 | GH1 | Growth Hormone 1 | 0.72773 | 0.91774 | 1.005162515 | 1.00516 |
| 12842660 | TSHB | Thyroid stimulating hormone beta | 0.999161 | 0.999934 | 1.000078006 | 1.00008 |
| 12844718 | TBX19 | T-Box 19 | 0.104204 | 0.458935 | 0.881026219 | -1.13504 |
| 12778522 | NEUROD1 | Neuronal differentiation 1 | 0.177765 | 0.56633 | 1.188588599 | 1.18859 |
| 12883226 | NR3C1 | Nuclear receptor subfamily 3 group C member 1 | 0.112395 | 0.473489 | 1.137451971 | 1.13745 |
| 12760857 | CRHR1 | Corticotropin releasing hormone receptor 1 | 0.106139 | 0.461876 | 1.19399706 | 1.194 |
| 12850526 | CRHR2 | Corticotropin releasing hormone receptor 2 | 0.724778 | 0.916408 | 1.028423571 | 1.02842 |
